# Genomic landscape of drug response reveals mediators of anthelmintic resistance

**DOI:** 10.1016/j.celrep.2022.111522

**Published:** 2022-10-18

**Authors:** Stephen R. Doyle, Roz Laing, David Bartley, Alison Morrison, Nancy Holroyd, Kirsty Maitland, Alistair Antonopoulos, Umer Chaudhry, Ilona Flis, Sue Howell, Jennifer McIntyre, John S. Gilleard, Andy Tait, Barbara Mable, Ray Kaplan, Neil Sargison, Collette Britton, Matthew Berriman, Eileen Devaney, James A. Cotton

**Affiliations:** 1 Wellcome Sanger Institute, Hinxton, Cambridgeshire CB10 1SA, UK; 2 Institute of Biodiversity Animal Health and Comparative Medicine, College of Medical, Veterinary and Life Sciences, University of Glasgow, Glasgow G61 1QH, UK; 3 Moredun Research Institute, Penicuik, Midlothian EH26 0PZ, UK; 4 Royal (Dick) School of Veterinary Studies, University of Edinburgh, Edinburgh EH25 9RG, UK; 5 Department of Infectious Diseases, College of Veterinary Medicine, University of Georgia, Athens, GA 30602, USA; 6 Department of Comparative Biology and Experimental Medicine, Host-Parasite Interactions Program, Faculty of Veterinary Medicine, University of Calgary, Calgary T2N 1N4, Canada

**Keywords:** *Haemonchus contortus*, helminth, anthelmintic resistance, genetic cross, forward genetics, ivermectin, levamisole, benzimidazole, genome-wide association, *cky-1*

## Abstract

Like other pathogens, parasitic helminths can rapidly evolve resistance to drug treatment. Understanding the genetic basis of anthelmintic drug resistance in parasitic nematodes is key to tracking its spread and improving the efficacy and sustainability of parasite control. Here, we use an *in vivo* genetic cross between drug-susceptible and multi-drug-resistant strains of *Haemonchus contortus* in a natural host-parasite system to simultaneously map resistance loci for the three major classes of anthelmintics. This approach identifies new alleles for resistance to benzimidazoles and levamisole and implicates the transcription factor *cky-1* in ivermectin resistance. This gene is within a locus under selection in ivermectin-resistant populations worldwide; expression analyses and functional validation using knockdown experiments support that *cky-1* is associated with ivermectin survival. Our work demonstrates the feasibility of high-resolution forward genetics in a parasitic nematode and identifies variants for the development of molecular diagnostics to combat drug resistance in the field.

## Highlights

• High genomic diversity differentiates susceptible and multi-drug-resistant helminths
• A forward genetic cross reveals QTLs that are specific to each of three drug classes
• Novel causal variants are defined for benzimidazole and levamisole anthelmintics
• *cky-1* expression is correlated with ivermectin resistance in three nematode species

## Graphical abstract

**Figure:**
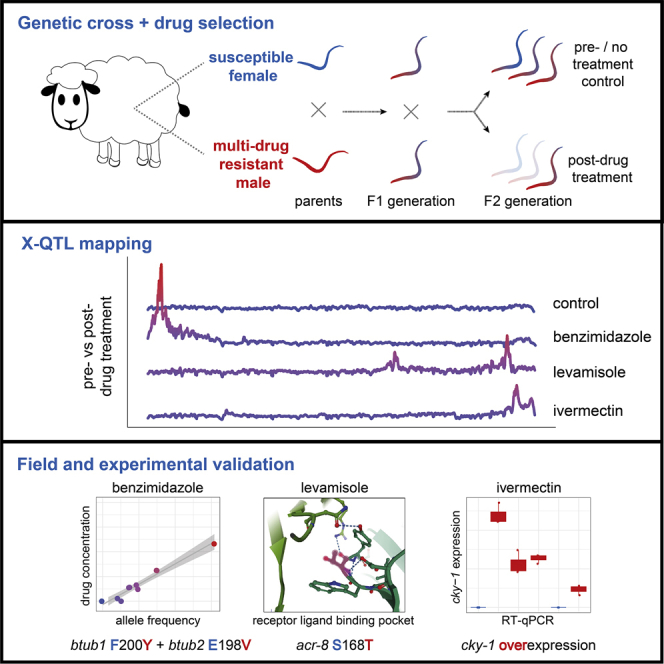

## Teaser

Doyle et al. describe a forward genetic cross between susceptible and multi-drug-resistant strains of the globally distributed parasitic worm *Haemonchus contortus*. Whole-genome analysis reveals loci associated with resistance to each drug class, and novel resistance alleles are validated for two classes.

## Introduction

Over a billion people and countless livestock and companion animals require at least annual treatment with drugs to control parasitic worm (helminth) infections. The evolution of resistance to these anthelmintic drugs, particularly by livestock-infective helminths, has been rapid and widespread; in many places, individual drug classes are now ineffective and some farms harbor parasites resistant to every major drug class (Kaplan and Vidyashankar, 2012). In Europe, gastrointestinal helminths of livestock are responsible for annual production losses of €686 million, of which €38 million is associated with anthelmintic resistance (Charlier et al., 2020). While most studies have focused on livestock parasites, and in particular, clade V (Blaxter et al., 1998) nematodes, drug resistance is also now a major concern in the treatment of the dog heartworm *Dirofilaria immitis* (a clade III species related to human filarial nematodes) (Bourguinat et al., 2015), and multi-drug resistance is now common in the United States in the dog hookworm *Ancylostoma caninum* (Jimenez Castro et al., 2019, 2021). The same drugs are also used to control related human-infective helminths, which are targeted by some of the most extensive preventive chemotherapy programs globally. Although less established in human-infective helminths, the emergence of widespread anthelmintic resistance—echoing the current global emergency around antimicrobial resistance—will have serious socioeconomic and welfare impacts on people infected with parasitic worms and derail hard-won progress toward the proposed elimination of helminths as a public health problem over the next decade (Montresor et al., 2020; World Health Organization, 2020).

Despite extensive efforts, the causal mutations and mechanisms of resistance in parasitic helminths remain largely unresolved. Many candidate "resistance genes" have been proposed; these candidates are primarily homologues of genes that confer resistance in the free-living model nematode *Caenorhabditis elegans* and are subsequently assayed for differences in genetic variation and/or gene expression in parasites that vary in their response to treatment (Doyle and Cotton, 2019; Hahnel et al., 2020; Kotze et al., 2014). A successful example of this approach is the identification of variants of β-tubulin that inhibit tubulin-depolymerization by benzimidazole-class anthelmintics (Driscoll et al., 1989; Roos et al., 1990). These variants, particularly at amino acid positions 167, 198, and 200 of β-tubulin isotype 1 (Ghisi et al., 2007; Kwa et al., 1993; Silvestre and Cabaret, 2002), have since been shown to be associated with resistance in many parasitic species for which benzimidazoles have been extensively used, and a number of these parasite-specific mutations have been functionally validated in *C. elegans* (Dilks et al., 2020; Kwa et al., 1995). However, these variants do not explain all phenotypic variation associated with resistance (Hahnel et al., 2018; Zamanian et al., 2018), and it is unknown if other variants contribute to benzimidazole resistance in parasitic species. For other drug classes, few candidate genes have been functionally validated or shown to be important in natural parasite populations. For example, concurrent mutation of three glutamate-gated chloride channels (*glc-1, avr-14, avr-15*) enables resistance to high concentrations of ivermectin by *C. elegans* (Dent et al., 2000), yet no strong evidence of selection on these channels in any parasitic species has been demonstrated to date. The many genes proposed may reflect that resistance is a complex, quantitative trait where similar resistance phenotypes can arise from variation in multiple loci. At the same time, resistance may be species- and/or population-specific and evolve independently under different selection pressures (Gilleard, 2013). However, most studies have focused on single or few candidate loci in small numbers of helminth populations that often differ in drug susceptibility and geographic origin, and therefore, some candidates are likely to have been falsely associated with resistance. Considering many helminth species are exceptionally genetically diverse (Berger et al., 2021; Doyle et al., 2017; Gilleard, 2006; Sallé et al., 2019), candidate gene approaches have limited power to disentangle causal variation from linked but unrelated background genetic variation.

Here we describe a genome-wide forward genetics approach using the parasitic nematode *Haemonchus contortus* to identify genetic variation associated with resistance to three of the most important broad-spectrum anthelmintic drug classes globally: macrocyclic lactones (ivermectin), imidazothiazoles (levamisole), and benzimidazoles (fenbendazole and thiabendazole). *H. contortus* is an economically important gastrointestinal parasite of livestock worldwide and one of only a few genetically tractable parasites used for drug discovery (Kaminsky et al., 2008; Preston et al., 2015), vaccine development (Bassetto and Amarante, 2015; Knox et al., 2003), and anthelmintic resistance research (Gilleard, 2013). Our approach has exploited a genetic cross between the susceptible MHco3(ISE) and multi-drug-resistant MHco18(UGA) strains of *H. contortus*, allowing us to investigate resistance in a natural host-parasite system while controlling for confounding genetic diversity that differentiates parasite strains. We performed quantitative trait locus (QTL) mapping to reveal drug-specific QTLs associated with resistance, which were independently validated using genome-wide variation in populations of *H. contortus* obtained from 10 US farms of known resistance phenotype and from more than 350 individual parasites sampled throughout the world where *H. contortus* is endemic (Doyle et al., 2020; Sallé et al., 2019).

## Results

### A genetic cross between susceptible and multi-drug-resistant worms reveals drug-specific QTLs after selection

**Figure 1:**
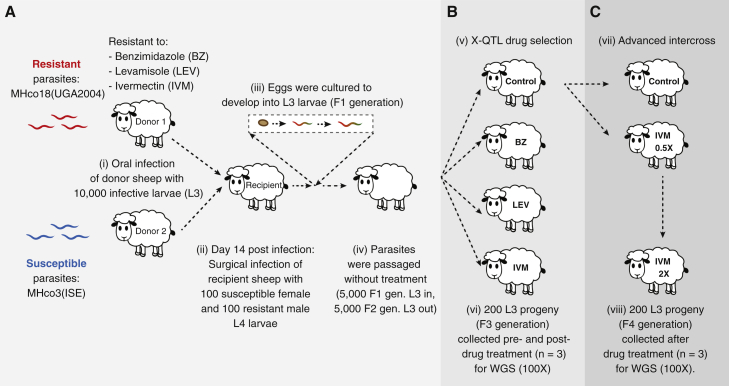
Outline of the genetic cross, X-QTL, and advanced intercross experiments (A) A genetic cross between the anthelmintic susceptible MHco3(ISE) and multi-drug-resistant MHco18(UGA) was used to map genetic loci associated with fenbendazole, levamisole, and ivermectin drug treatment. (B) An X-QTL experiment was performed on the F2 generation exposed to fenbendazole, levamisole, or ivermectin or not treated. (C) An advanced intercross experiment using the F3 generation was subjected to a half-dose followed by a double-standard dose of ivermectin. For both the X-QTL and advanced intercross experiments, pools of L_3_ (n = 200) were collected pre- and post-treatment from drug-exposed and time-matched untreated controls, performed in triplicate (Figure S1). Whole-genome sequencing was performed, and genetic diversity between pre- and post-treatment was compared.

To identify drug-resistance associated QTLs, we have used a genetic cross between drug-susceptible and multi-drug-resistant strains of *H. contortus* together with an eXtreme Quantitative Trait Locus (X-QTL) (Burga et al., 2019; Chevalier et al., 2014) drug selection and analysis framework (Figure 1A). Drug selection was performed on pools of F3-generation progeny from F2 adults treated *in vivo* using one of fenbendazole, levamisole, or ivermectin (Figures 1B and S1); parasites were sampled pre- and post-treatment followed by whole-genome sequencing (Table S1). A sampling-time matched but untreated control was also included for comparison.

**Figure 2:**
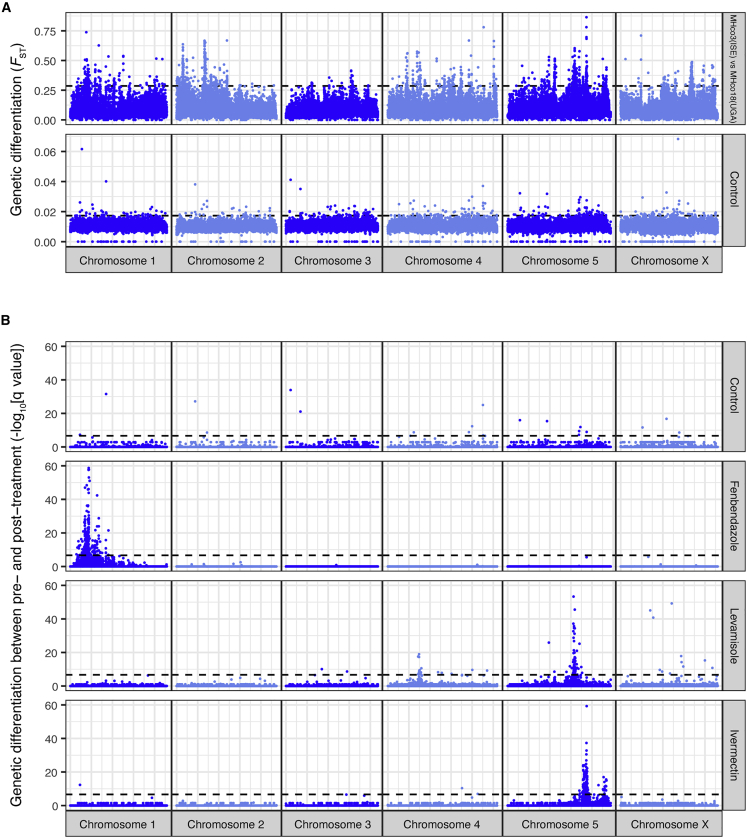
A genetic cross followed by drug selection reveals discrete QTLs associated with each anthelmintic drug class (A) Genome-wide comparison of susceptible MHco3(ISE) and multidrug-resistant MHco18(UGA) parental strains revealed broad-scale genetic differentiation (*F*_ST_) on all chromosomes. In contrast, after the genetic cross, these signals of differentiation are lost in an untreated control (time-matched samples to the drug-treated groups). The dashed line represents the mean *F*_ST_ + 3 standard deviations. (B) Comparison of genome-wide differentiation between F3 generation pooled infective-stage larvae (L_3_, n = 200) sampled pre- and post-treatment revealed distinct genomic regions or QTLs associated with fenbendazole, levamisole, and ivermectin drug treatment. In all plots, each point represents the -log_10_ q value from the *Z* score distribution of mean genetic differentiation (*F*_ST_) from three biological replicates per 5 kb sliding window throughout the genome. The dashed line represents the Bonferroni genome-wide level of significance (α = 0.05, n = 56,476 windows). See Figure S1 for genome-wide replicate data of the drug selection experiments.

The susceptible and resistant *H. contortus* strains used to initiate the cross were highly genetically differentiated throughout their genomes (Figure 2A MHco3(ISE) versus MHco18(UGA); mean *F*_ST_ = 0.089 ± 0.066 SD; n = 16,794,366 single nucleotide variant sites), typical of two parasite strains sampled from different continents (Doyle et al., 2019a; Sallé et al., 2019). The high degree of within-strain diversity and genome-wide genetic divergence is controlled by admixture in the F1 generation of the cross, where susceptible and resistant alleles are expected to segregate in the absence of selection, and genetic recombination will break down linked genetic variation that differentiates the parental strains. This is evident by significantly lower genome-wide genetic differentiation in the F3 untreated population (genome-wide mean *F*_ST_ = 0.012 ± 0.004) and the absence of discrete peaks of high genetic differentiation (Control; Figures 2A and 2B). In contrast, after each drug treatment, discrete QTLs that differ between each drug class were revealed (Figure 2B): after fenbendazole treatment, we identified a major QTL on chromosome 1; after levamisole, two QTLs on chromosomes 4 and 5; and after ivermectin, a major QTL on chromosome 5 and minor QTL on chromosome 5.

### Variation at β-tubulin isotype 1 and a novel isotype 2 variant is associated with high levels of benzimidazole resistance

**Figure 3:**
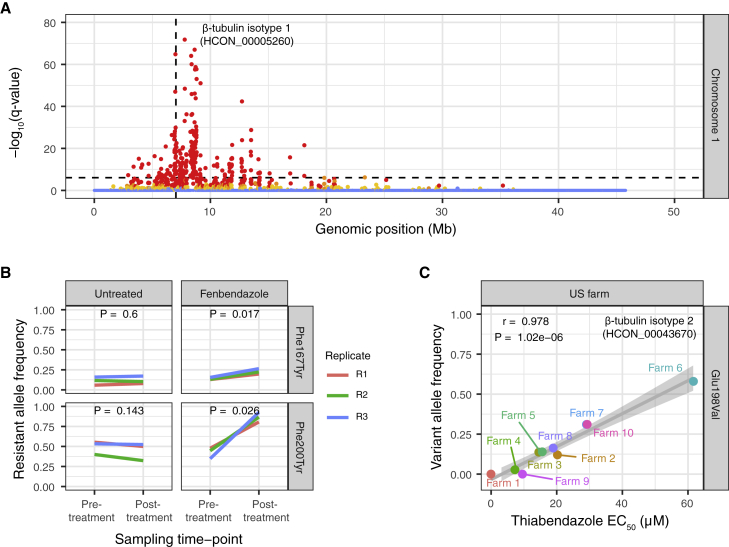
Characterization of QTLs associated with benzimidazole resistance (A) Chromosome-wide genetic differentiation between pre- and post-fenbendazole treatment on chromosome 1. Each point represents the -log_10_ q value from the *Z* score distribution of mean genetic differentiation (*F*_ST_) from three biological replicates per 5 kb sliding window throughout the genome; points are colored based on the concordance of individual replicates indicated by none (blue), 1 of 3 (yellow), 2 of 3 (orange), or all 3 (red) above the genome-wide threshold. The horizontal dashed line represents the Bonferroni genome-wide level of significance (α = 0.05, n = 56,476 windows). (B) Allele frequency change at Phe167Tyr and Phe200Tyr variant positions of β-tubulin isotype 1 pre- and post-treatment, including untreated time-matched control. Colored lines represent individual biological replicates (n = 3). p values are calculated using pairwise t tests of allele frequency by sampling time point (i.e., pre- and post-treatment). (C) Correlation between thiabendazole EC_50_ concentration (μM, measured using the *in vitro* DrenchRite assay) and Glu198Val variant frequency of β-tubulin isotype 2 (HCON_00043670) on US farms. Pearson’s correlation (r) and associated p value together with the trendline and standard error of the linear regression are shown.

The β-tubulin isotype 1 (HCON_00005260) gene and, in particular, non-synonymous changes at coding positions 167, 198, and 200 have been widely associated with benzimidazole resistance in *H. contortus* (Ghisi et al., 2007; Kwa et al., 1994; Roos et al., 1990; Silvestre and Cabaret, 2002) and other nematodes frequently exposed to benzimidazole treatment (Avramenko et al., 2019; Dilks et al., 2020; Furtado et al., 2014; Martínez-Valladares et al., 2020). After fenbendazole selection, a single broad QTL was found on chromosome 1 (Figure 3A) containing the β-tubulin isotype 1 locus. Within this gene, we identified a significant increase in the frequency of a Phe200Tyr variant (a phenylalanine [reference susceptible variant] to tyrosine [resistant variant] substitution at position 200) from pre- to post-treatment and relative to untreated controls (Figure 3B; p = 1.7 × 10^−26^, genome-wide Cochran-Mantel-Haenszel (CMH) test). We also identified a small increase in the frequency of the Phe167Tyr variant (*freq*_pre-treatment_ = 0.14 to *freq*_post-treatment_ = 0.20); however, no variation was found at the Glu198 position. We note that Phe200Tyr was not the most significantly differentiated variant between pre- and post-treatment in the region; 817 polymorphic sites, including eight non-synonymous variants, were identified on chromosome 1 with a p value equal to or lower than the variant causing the Phe200Tyr substitution, highlighting the challenge of identifying causal mutations in a highly genetically diverse species even under the controlled conditions of the genetic cross. Nonetheless, considering the previous association with benzimidazole resistance, we conclude that the Phe200Tyr variant is the primary driver of phenotypic resistance in the X-QTL population.

Despite the identification of a known candidate gene, the genetic structure of the fenbendazole-associated QTL was surprising, given the β-tubulin isotype 1 gene was not in the middle of the QTL as expected but was found on the right edge of an approximately 3 Mb region of genetic differentiation extending downstream of the locus. The relevance of this extended genetic structure within the QTL is unclear. Although a QTL surrounding the β-tubulin isotype 1 locus was present in the US farms that are phenotypically resistant to benzimidazoles (Figure S2), the additional downstream region of differentiation was absent. The broader QTL from the genetic cross may reflect an artifact of the cross due to linkage disequilibrium with the Phe200Tyr mutation; however, a comparison of this genetic signal against the genetic map (Doyle et al., 2018) suggests that recombination rates are not unusual within this region compared with flanking regions. Larger structural variation may exist between the two strains, such as a deletion or large-scale inversion of chromosome sequence; however, no unusual sequence features such as low coverage could explain this signal. Long-read sequencing or long-molecule data such as high-throughput chromatin conformation capture (HiC) may provide further insight into potential cryptic structural variation. Alternatively, this broader region may represent the selection of additional loci that require further investigation. For example, it is notable that two of the eight non-synonymous variants were found in cathepsin B-like cysteine proteinases (HCON_00005790 and HCON_00005960), which have previously been implicated in benzimidazole-mediated damage of the intestinal cells of *H. contortus* (Jasmer et al., 2000; Shompole and Jasmer, 2001).

*H. contortus* has multiple β-tubulin genes (Saunders et al., 2013) and deletion of the β-tubulin isotype 2 gene (HCON_00043670) on chromosome 2 has previously been associated with increased levels of resistance beyond that of mutations in the isotype 1 gene alone (Kwa et al., 1993). Here, we found no evidence of deletions in isotype 2. However, a minor but non-significant increase in genetic differentiation between pre- and post-treatment populations was found, and a Glu198Val variantat a homologous site to a known resistance variant in isotype 1 was present at a low frequency in the genetic cross (*freq*_pre-treatment_ = 0.260 to *freq*_post-treatment_ = 0.323; not significant by CMH). However, the Glu198Val variant did vary in frequency between US farm populations (Figures S2 and S3) and was significantly correlated (r = 0.978, p = 1.02 × 10^−6^; Pearson’s correlation) with half maximal effective concentration (EC_50_) values for thiabendazole resistance (Figure 3C). The variance observed in EC_50_ among resistant farm populations was not associated with the frequency of Phe200Tyr of isotype 1, as this variant was already at high frequency in resistant populations (Figure S4). These data suggest that once the isotype 1 Phe200Tyr variant has reached near fixation in the population, the Glu198Val variant of isotype 2 mediates higher levels of thiabendazole resistance than conferred by the Phe200Tyr variant alone. As such, this novel allele in β-tubulin isotype 2 should be considered as a genetic marker for benzimidazole resistance.

In addition to the association with benzimidazole resistance, it has been suggested that the β-tubulin isotype 1 Phe200Tyr variant in *H. contortus* (Eng et al., 2006; de Lourdes Mottier and Prichard, 2008; Santos et al., 2017) and also at an equivalent variant site in a β-tubulin gene in the human-infective filarial nematode *Onchocerca volvulus* (Osei-Atweneboana et al., 2012) is associated with ivermectin resistance. Here we found no evidence of selection on either the Phe167Tyr or Phe200Tyr variants (or any variant found in the region) in X-QTL analyses of ivermectin treatment (Figure S5A), nor any correlation with ivermectin EC_50_ on the US farms (Figure S5B). These data reaffirm that mutations in β-tubulin isotype 1 are specific to benzimidazole resistance.

### Levamisole selection implicates acetylcholine receptors, including a novel *acr-8* variant, with resistance

**Figure 4:**
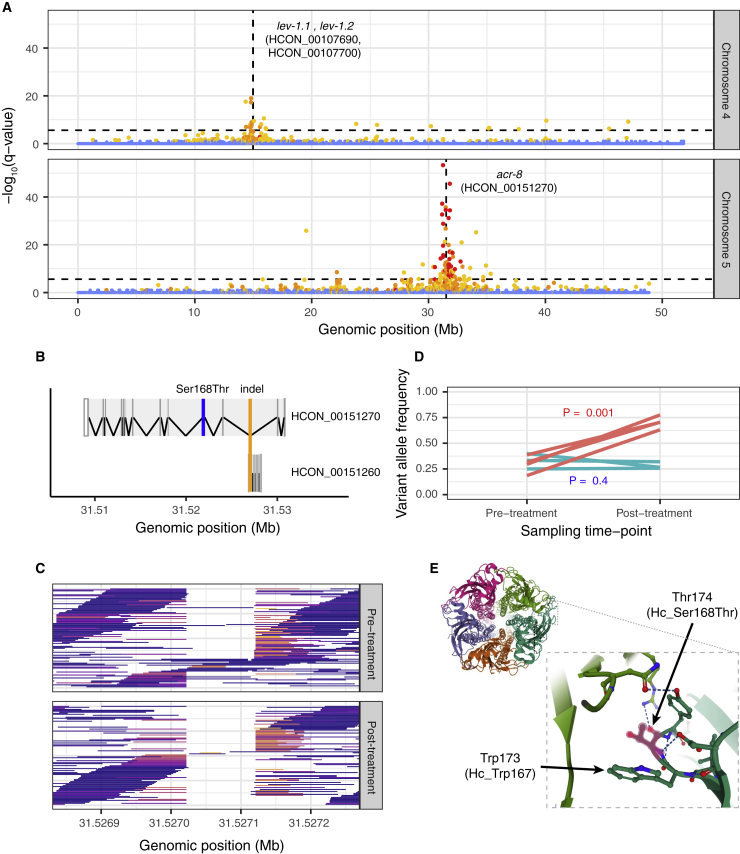
Characterization of QTLs associated with levamisole resistance (A) QTLs between pre-treatment and levamisole-treated parasites on chromosome 4 (top) and chromosome 5 (bottom). Each data point represents the -log_10_ q value from the *Z* score distribution of mean genetic differentiation (*F*_ST_) from three biological replicates per 5 kb sliding window throughout the genome; points are colored based on the concordance of individual replicates indicated by none (blue), 1 of 3 (yellow), 2 of 3 (orange), or all 3 (red) above the genome-wide threshold. The horizontal dashed line represents the Bonferroni genome-wide level of significance (α = 0.05, n = 56,476 windows). (B) Gene model for *acr-8* (top; HCON_00151270) and a cuticle collagen (bottom; HCON_00151260), highlighting the position of the overlapping *acr-8*/levamisole-associated indel (orange line) and the Ser168Thr variant (blue line) of *acr-8*. (C) Visualization of sequencing reads supporting the *acr-8* intronic indel. Mapped reads are colored to reflect the degree to which they have been clipped to allow correct mapping in the presence of the deletion, i.e., reads that have not been clipped are blue, whereas reads that are moderate to highly clipped are colored red to yellow, respectively. (D) Comparison of Ser168Thr variant frequency between pre- and post-levamisole treatment (red) and time-matched untreated controls (green). Each line represents a biological replicate (n = 3). (E) Structure of the pentameric Cys-loop acetylcholine receptor of *Torpedo marmorata* (Protein DataBase ID: 4AQ9), one of the few species from which the receptor’s structure has been resolved (Unwin and Fujiyoshi, 2012). The Trp[Ser/Thr]Tyr motif is highly conserved among the clade V nematodes (Figure S6) and the distantly related alpha subunit of *T. marmorata*; Thr174, the homologous position of the *H. contortus* Hc_Ser168Thr variant of *acr-8,* lies within the acetylcholine binding pocket at the interface of the alpha and gamma subunits and adjacent to Trp173 (*H. contortus* Hc_Trp167), a residue essential for ligand binding.

The anthelmintic activity of levamisole is due to its antagonistic effect on nematode nicotinic acetylcholine receptors (Martin et al., 2012), and resistance in *C. elegans* is typically associated with variation in subunits of these receptors or other accessory proteins that contribute to acetylcholine-mediated signaling (Fleming et al., 1997). Here we identified two major QTLs, one on chromosome 4 that contained a tandem duplication of the acetylcholine receptor subunit β-type *lev-1* (HCON_00107690 and HCON_00107700) and one on chromosome 5 that contained the acetylcholine receptor subunit *acr-8* (HCON_00151270) (Figure 4A).

The *H. contortus acr-8* gene has long been implicated in levamisole resistance; a truncated isoform of *acr-8* containing the two first exons and a part of intron 2 (previously called *Hco-acr-8b*) (Fauvin et al., 2010) and subsequently a 63-base pair (bp) indel between exons 2 and 3, have been associated with resistance based on their presence in several levamisole-resistant isolates (Barrère et al., 2014). However, the functional consequence of these variants in mediating levamisole resistance *in vivo* is not yet clear. Here, we identified two larger deletion variants spanning 31,527,022 to 31,527,119 (97 bp) or 31,527,121 (99 bp) within the *acr-8* gene (Figure 4B; orange line) on chromosome 5 that increased in frequency from 73.47% in the pre-treatment population to 86.58% after levamisole treatment (Figure 4C; paired t test across replicates, p = 0.1). However, the *acr-8* indel was present in the levamisole susceptible parental MHco3(ISE) strain (59.05%) and was present at only a slightly higher frequency in the resistant MHco18(UGA) strain (63.55%). Thus, these data argue that the *acr-8* indel is a poor marker of levamisole resistance.

We did, however, identify a non-synonymous variant (Ser168Thr) in *acr-8* (Figure 4B; blue line) that was strongly correlated with resistance across multiple datasets. In the X-QTL analyses, Ser168Thr increased to a high frequency after drug selection in the F2 generation (Figure 4D; position 31,521,884; genome-wide CMH: p = 1.6 × 10^−15^). It was also found at a high frequency in the US field population with the highest levamisole drug resistance phenotype (Farm 7; *freq*_Ser168Thr_ = 0.64). This association was supported by global diversity data of *H. contortus* (Sallé et al., 2019), where Ser168Thr was fixed in parasites from the Kokstad (KOK; South Africa) population (*freq*_Ser168Thr_ = 1.0; n = 4), the only population with confirmed levamisole resistance in that study, but absent in all other populations analyzed. The identification of Ser168Thr prompted us to look beyond *H. contortus*; a reanalysis of levamisole resistance in closely related clade V parasitic nematode *Teladorsagia circumcincta* (Choi et al., 2017) revealed a homologous non-synonymous variant at high frequency in resistant parasites (Ser140Thr in Cont419:G75849C; *freq*_Ser140Thr_ = 0.972), which was absent in the susceptible population to which it was compared. The serine residue was highly conserved among clade V nematodes (Figure S6), particularly among the parasite species, whereas in the free-living *Caenorhabditis* spp., threonine is encoded at this position. In *C. elegans, acr-8* is genetically and functionally distinct from *acr-8* of parasitic nematodes and is not a component of the native levamisole receptor (Hernando et al., 2012); the *C. elegans* functional homolog *lev-8*, which can be transgenically substituted by *H. contortus acr-8* to produce a functional receptor (Blanchard et al., 2018), does encode a serine at this homologous position. The *H. contortus* ACR-8 Ser168Thr variant lies immediately downstream of the Cys-loop domain within the ligand-binding pocket and is immediately adjacent to a highly conserved tryptophan residue essential for ligand binding (Lynagh and Pless, 2014; Williams et al., 2009) (Figure 4E). Importantly, key residues downstream of the conserved tryptophan have previously been shown to influence levamisole sensitivity of closely related receptor subunits (Rayes et al., 2004). Thus, we hypothesize that the Ser168Thr variant facilitates a change in molecular interactions within the binding pocket of ACR-8, resulting in a decreased sensitivity to levamisole.

The identification of *lev-1* genes within the chromosome 4 QTL is compelling, with three intronic variants of *lev-1* (top variant position 14,995,062 in HCON_00107700; p = 1.7 × 10^-20^; CMH test) among the top 10 most differentiated SNPs on this chromosome. However, it remains unclear what effect variation in the *lev-1* genes has on levamisole resistance. Although multiple non-synonymous variants were also identified (seven and three variants for HCON_00107690 and HCON_00107700, respectively), none were predicted to cause high-effect changes in the protein sequence and exhibited only relatively minor shifts in allele frequency upon levamisole treatment. In *C. elegans*, several dominant resistant variants of *lev-1* have been described (not found in the data described here); however, *lev-1* can be lost without affecting the function of the receptor (Fleming et al., 1997). Analyses of *lev-1* expression and genetic variation may be required to determine its role in levamisole resistance. Close to the *lev-1* genes and toward the center of the QTL, four of the top 10 variants in chromosome 4 were found in HCON_00107560 (top non-synonymous variant: Arg934His at position 14,781,344; p = 1.0 × 10^−21^; CMH test), an ortholog of *C. elegans kdin-1*. Highly conserved with mammalian orthologs (Kong et al., 2001), *kdin-1* has been shown to co-localize with acetylcholine receptors at rat neuromuscular junctions during development (Luo et al., 2005) where, via a PDZ domain, it participates in the coordination of signaling components including ion channels and neurotransmitters. The precise role of HCON_00107560 in *H. contortus* or *kdin-1 in C. elegans* remains unknown; however, its putative association with levamisole response here warrants further investigation.

Signals of selection on two components of the pentameric acetylcholine receptor prompted us to look for selection on the remaining subunits. Although the expression of *unc-63* (HCON_00024380) and *unc-29.3* (HCON_00003520) mRNAs have been previously shown to be significantly reduced in the larvae of the resistant MHco18(UGA) strain (Williamson et al., 2011), we found no evidence of selection on the regions of the genome containing these genes.

### A resolved ivermectin QTL implicates *cky-1* as a novel mediator of resistance

Ivermectin is a critically important broad-spectrum drug used to control several human- and veterinary-infective helminths worldwide and is also widely used as an acaricide targeting ticks and mites. We recently identified an ∼5 Mb QTL associated with ivermectin resistance from 37 to 42 Mb on chromosome 5 from the analysis of a backcross experiment (Doyle et al., 2019a; Redman et al., 2012), and subsequently, we identified evidence of selection in the same chromosomal region in ivermectin-resistant field populations from Africa and Australia (Sallé et al., 2019). Although the introgression region from the backcross was broad (Doyle et al., 2018), the genetic architecture of the QTL was consistent with a single dominant variant driving resistance, and we confirmed that most candidate genes previously proposed to be associated with resistance were not under direct ivermectin selection. However, we could not confidently identify any novel candidate driving mutation among the ∼360 genes within the region (Doyle et al., 2019a).

**Figure 5:**
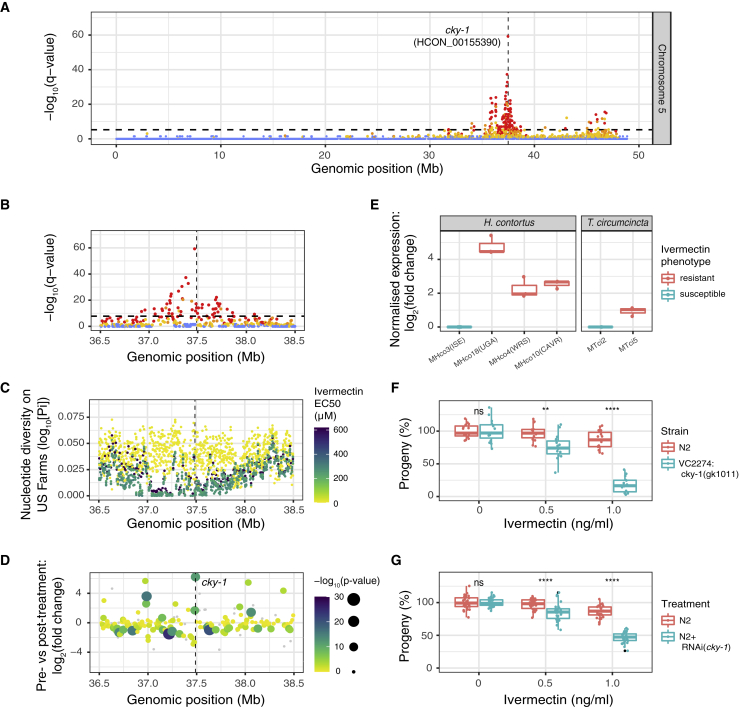
Characterization of the major QTL associated with ivermectin resistance (A) QTL between pre- and post-ivermectin treatment on chromosome 5. Each data point represents the -log_10_ q value from the *Z* score distribution of mean genetic differentiation (*F*_ST_) from three biological replicates per 5 kb sliding window throughout the genome; points are colored based on the concordance of individual replicates indicated by none (blue), 1 of 3 (yellow), 2 of 3 (orange), or all 3 (red) above the *F*_ST_ genome-wide threshold. The horizontal dashed line represents the Bonferroni genome-wide level of significance (α = 0.05, n = 56,476 windows). A magnified aspect of the main chromosome 5 QTL, highlighting (B) -log_10_ q value of *F*_ST_ in the X-QTL cross, and (C) nucleotide diversity (Pi) on US farms, where each farm is colored by the degree of ivermectin resistance (EC_50_) measured by larval development assays. In (A), (B), (C), and (D), the position of *cky-1* is indicated by the vertical dashed line. (D) Reanalysis of RNA-seq data from (Laing et al., 2022), highlighting the position of *cky-1* in the QTL and overexpression after treatmenI(E) RT-qPCR analysis of *cky-1* expression in *H. contortus* and *T. circumcincta* strains that differ in their ivermectin resistance phenotype. Data represent log_2_-transformed expression normalized to actin or GAPDH control genes for *H. contortus* and *T. circumcincta,* respectively, from three independent experiments. Downregulation of *cky-1* expression in *C. elegans* by either (F) a balanced deletion or (G) RNAi-knockdown increases ivermectin sensitivity relative to the control N2 strain, based on developmental assays (n = 3 independent experiments) measuring the percentage of progeny surviving to adulthood relative to DMSO controls. In (F) and (G), each point represents an independent treatment condition, normalized to a DMSO control without ivermectin. A Kruskal-Wallis test was used to determine whether treatment condition differed from untreated control, where ns = not significant, ^∗^p < 0.05, ^∗∗^p < 0.01, and ^∗∗∗∗^p < 0.0001. Boxplots in (E), (F), and (G) show the median, 25th, and 75th percentiles of the data. The whiskers extend 1.5 times the inter-quartile range.

Here, we confirm the QTL within the previously implicated chromosome 5 region at ∼37.5 Mb (Doyle et al., 2019a; Redman et al., 2012) but with significantly increased resolution (Figure 5A). We have narrowed the genetic association to approximately 300 kb wide (region: ∼37,250,000–37,550,000) based on the region of highest differentiation between independently replicated pre- and post-treatment X-QTL samples (Figure 5B). This region was also highly differentiated between pre-treatment larvae and adult male worms that survived ivermectin treatment *in vivo* (Figure S7A and S7B) and between larvae that survived treatment with an EC_75_ dose of ivermectin and those sensitive to an EC_50_ dose *in vitro* (Figure S7C and S7D)*.* Together, these results confirm that this locus is under selection and mediates resistance in both the parasitic stages *in vivo* and free-living stages *in vitro*. Finally, this was the only region in the genome where increased levels of ivermectin resistance (i.e., EC_50_) were associated with a loss of genetic diversity in moderately or highly resistant field populations relative to susceptible populations (Figure 5C), consistent with a selective sweep in response to ivermectin-mediated selection.

The main chromosome 5 QTL contained 25 genes and included an expansion of protein kinases (8 of 21 genes present in the genome with the InterPro identifier IPR015897), some of which had the highest statistical association with resistance; for example, HCON_00155240 (intronic position 37,336,132, p = 3.3 × 10^−13^; position 37,335,944, p = 1.2 × 10^−12^) and HCON_00155270 (intronic position 37,343,439, p = 1.0e-10). However, these protein kinases are novel leads with no previous association with drug resistance, and a lack of functional orthologs and observed gene expansion made it difficult to further infer and test a role for these genes in ivermectin resistance.

Toward the middle of the QTL, we identified *cky-1* (HCON_00155390; positions 37,487,982 - 37,497,398) as a new mediator of resistance, based on several lines of evidence. In the X-QTL data, *cky-1* contained eight moderately to highly differentiated significant non-synonymous variants (top variant: position 37,497,061 [Ser583Pro], p = 9.6 × 10^−9^; CMH test). In a complementary study, we showed that *cky-1* was the only gene in the region significantly upregulated in both males and females of the resistant MHco18(UGA) isolate relative to MHco3(ISE) and was one of the most upregulated genes genome-wide (Laing et al., 2022) (see Figure 5D zoomed-in reanalysis of RNA sequencing (RNA-seq) data from Laing et al.). In this study, RT-qPCR of *cky-1* in three unrelated, genetically distinct ivermectin-resistant *H. contortus* strains revealed significant overexpression of *cky-1* in ivermectin-resistant relative to the sensitive MHco3(ISE) strain (Figure 5E). This observation was replicated between sensitive and ivermectin-resistant strains of the related parasite, *T. circumcincta* (Figure 5E). To explore the role of *cky-1* further, we assayed the developmental phenotypes *of C. elegans*, known to be perturbed by ivermectin exposure (Dent et al., 1997), to test the role of differential expression of *cky-1* on the resistant phenotype in the presence of ivermectin. While complete knockout of *cky-1* was non-viable, both a balanced deletion (VC2274) (Figure 5F) and RNAi knockdown (Figure 5G) of *cky-1* increased the sensitivity of *C. elegans* to ivermectin relative to the ivermectin-susceptible N2 strain. There remains the possibility that knocking down *cky-1* may reduce worm fitness, which could theoretically produce a sensitivity phenotype that is not specific to ivermectin, and this warrants further study. However, collectively, these data support the hypothesis that the level of *cky-1* expression is associated with the ivermectin resistance phenotype in nematodes.

Two additional, less prominent QTLs were also identified after ivermectin treatment on chromosome 5 at ∼46 Mb and on chromosome 2 at ∼3 Mb. The minor chromosome 5 QTL was identified as a candidate region associated with resistance in the backcross (Doyle et al., 2019a); however, we did not have the statistical power to differentiate it from the main QTL in that experiment. Here, the QTL appeared to segregate independently of the prominent 37.5 Mb peak, providing more robust evidence of a second resistance-conferring variant on chromosome 5. Two genes, *avr-15* (HCON_00161180; 45,134,921-45,149,462) and *pgp-11* (HCON_00162780; 47,243,601-47,259,265), that have previously been associated with ivermectin resistance (Dent et al., 1997; Janssen et al., 2013) were identified. Here, both genes were found on the boundaries of the broad QTL that ranged from approximately 45 to 47.5 Mb spanning 221 genes in total, and while they do contain statistically significant non-synonymous variants (three and five for *avr-15* and *pgp-11*, respectively), the differences in variant frequency between pre- and post-treatment are relatively minor compared with other variants within the region, leading us to conclude that they are unlikely to be the direct target of selection here. Although the main chromosome 5 QTL at 37.5 Mb was present in all selection experiments with ivermectin, the secondary QTLs were variable between replicates and experiments.

The chromosome 2 QTL (region: 2,992,500–3,267,500; most prominent in Figure S1 X-QTL replicate 1) contained two previously described candidate genes, *osm-1* (HCON_00035760) and *che-11* (HCON_00035880), that have been shown to confer resistance either directly (Page, 2018) or additively with other variants (Dent et al., 2000) in *C. elegans*. Neither gene contained high effect variants; however, both contained a number of non-synonymous variants, including three moderately differentiated variants between pre- and post-treatment in *osm-1* (pos. 3,109,316 [Gln1540Pro], p = 8.444 × 10^−5^; pos. 3,109,310 [Val1542Gly], p = 8.337 × 10^−6^; pos. 3,109,002 [Glu1577Asp], p = 4.936 × 10^−4^) and four variants with significant but less differentiated variants in *che-11*. A glutamate transporter family protein *glt-5* (HCON_00035710) was also identified; however, there was no evidence of selection at this locus.

To improve the resolution of the QTLs identified and, perhaps, refine the variants under selection to those that specifically confer high resistance levels, we performed a follow-up experiment to the X-QTL by treating sheep infected with the F3 generation of the cross with a half-standard dose (0.1 mg/kg), followed by a subsequent double standard-dose treatment (0.4 mg/kg) (Figure 1C; Advanced Intercross). The rationale was first to identify low-effect variants (responding to the half-dose treatment), then select a subset of variants that conferred resistance at high doses. We consistently detected the main chromosome 5 QTL in these experiments but not the less prominent chromosome 2 QTL. We also detected at least three additional QTLs that appear in at least one but not all three replicates (Figure S8). One hypothesis is that these QTLs represent stochastic noise unrelated to drug exposure, evidenced by the fact that they only appear in a single replicate (apart from the chromosome 2 peak that does replicate from the X-QTL analyses). Alternatively, these discrete QTLs may provide evidence of selection on different genes in different sub-populations that contribute to the resistance phenotype (i.e., additively to provide additional levels of resistance and/or to compensate for fitness costs associated with variation needed for resistance) together with the necessary and sufficient causative allele on chromosome 5. If the latter is correct, these data allow us to speculate that underdosing of drug treatment to control parasites like *H. contortus* (represented here by the half-standard dose treatment) may “prime” different loci providing low-level but improved fitness in the presence of the drug, and may, in turn, lead to different genome-wide signatures of selection in field populations that have been exposed to different control strategies.

## Discussion

Anthelmintics are a critically important tool for controlling parasitic worm infections in humans and animals worldwide, and this is likely to remain true for the foreseeable future. However, this control paradigm is threatened by the emergence and spread of anthelmintic-resistant parasites. Here we demonstrate an efficient approach to map multiple drug resistance-conferring loci for three of the most important anthelmintic drugs in the globally distributed and genetically tractable parasitic nematode, *H. contortus*. We have identified novel variants and loci likely involved in resistance to each of these drug classes; these include the β-tubulin isotype 2 Glu198Val variant correlated with thiabendazole resistance in field populations, the *acr-8* Ser168Thr variant associated with levamisole resistance in both the cross and field populations of *H. contortus*, and *cky-1* as a novel candidate gene that mediates ivermectin response. Our approach was validated by identifying QTL and variants previously associated with drug resistance; for example, the β-tubulin isotype 1 Phe200Tyr variant associated with fenbendazole resistance and the *acr-8* indel variant associated with levamisole resistance. However, for the latter, we provide evidence against the indel being a reliable marker of resistance. Finally, we note an absence of many previously proposed ivermectin-associated candidate genes in the QTL described, highlighting both the limitation of candidate gene approaches and the power of genome-wide forward-genetic strategies to robustly identify regions of the genome containing known and novel mediators of resistance (Doyle and Cotton, 2019).

Functional genetic evidence from expression and knockout experiments has allowed us to explicitly test the role of our proposed candidate in the refined major ivermectin QTL on chromosome 5, the NPAS4 ortholog *cky-1*. This gene encodes an activity-dependent basic-Helix-Loop-Helix (bHLH)-PAS family transcription factor shown in mammals to regulate the excitation/inhibition balance upon neuronal activation to limit excitotoxicity (Spiegel et al., 2014) and during the development of inhibitory synapses to control the expression of activity-dependent genes (Lin et al., 2008). It is yet to be determined if this is a conserved molecular function in nematodes; however, it is tempting to speculate that the hyperexcitability as a result of induced activation of ion channels by ivermectin at the neuromuscular junction is, at least in part, controlled by a “retuning” of the excitation/inhibition balance to limit ivermectin-mediated effects. The role of *cky-1* in ivermectin resistance is supported by the following: (1) genetic differentiation between susceptible and resistant strains around this locus relative to genome-wide variation that is replicated in geographically and genetically diverse strains here and elsewhere (Baltrušis et al., 2022; Doyle et al., 2019a; Rezansoff et al., 2016; Sallé et al., 2019), (2) the presence of non-synonymous variants that are highly differentiated before and after treatment, (3) increased gene expression of *cky-1* in resistant strains relative to a susceptible strain (supported by genome-wide RNA-seq [Laing et al., 2022]), and (4) knockdown of the *C. elegans* ortholog leading to hypersensitivity to ivermectin. We acknowledge that overexpression of *cky-1* in *C. elegans* does not recapitulate the high levels of ivermectin resistance seen in *H. contortus* or, for example, by concurrent mutation of glutamate-gated chloride channels in *C. elegans* (Dent et al., 2000); while this may argue against *cky-1* as a universal mediator of resistance, it likely reflects the challenge of using a heterologous expression system in which there is an assumption that the biology (and, therefore, response to treatment) is concordant between the free-living and parasitic species, and/or may reflect the multigenic nature of ivermectin resistance in different species (Evans et al., 2021; Streit, 2021; Wit et al., 2021). Given the lack of an obvious causal non-synonymous variant, we hypothesize that a non-coding variant that influences the expression of *cky-1* is under selection in resistant strains of *H. contortus*; however, such variants are difficult to validate without genotype and transcriptional phenotype data from a large number of individual worms.

It is broadly accepted that the mode of action of ivermectin is on ligand-gated ion channels, and ivermectin resistance has been associated with variants in glutamate-gated channels (Laing et al., 2017). Concurrent mutation of a number of these channels (*glc-1, avr-14,* and *avr-15*) confers high-level resistance in *C. elegans* (Dent et al., 2000) and selection on at least one of these channels (*glc-1*) in wild strains (Ghosh et al., 2012) has been demonstrated. We find no evidence to suggest that genetic variation in these channels confers ivermectin resistance in *H. contortus*. Transcriptional changes in the expression of these channels in resistant, relative to drug-susceptible, parasite strains have been demonstrated previously; for example, glutamate-gated chloride channel subunits (*glc-3, glc-5*), as well as p-glycoprotein ABC transporters (*pgp-1, pgp-2, pgp-9*) (Williamson et al., 2011) in the MHco18(UGA) strain. Similarly, a *pgp-9* copy number variant was associated with ivermectin resistance in a genetic cross and bulk segregant experiment in the related nematode *T. circumcincta* (Choi et al., 2017), while transgenic overexpression of the equine parasitic nematode *Parascaris univalens pgp-9* (a clade III species) modulated ivermectin sensitivity in *C. elegans* (Gerhard et al., 2021). However, none of these genes were identified in regions of differentiation after treatment in this study, suggesting these genes were not the direct target of selection in *H. contortus*. However, we cannot exclude that variation in expression of these genes may be a downstream response to selection on a transcriptional regulator such as *cky-1*.

The use of genetic crosses, in which the genetics of the parasites can be controlled, is the ideal way to generate populations of individuals in which the relationship between genotype and phenotype can be assayed. Our approach relied on selecting populations of parasites using drug treatment; however, advances are still required to improve the phenotyping of resistance in individual parasites. The ability to do so would enable more sophisticated genetic approaches to unravel the role of the minor signatures of selection we observe in this experiment and improve our understanding of the molecular basis of drug resistance phenotypes. Recent advances in single larvae whole-genome sequencing (Doyle et al., 2019b) and low-input RNA-seq (Sun et al., 2021), even at single-cell resolution (Diaz Soria et al., 2020), now provide the tools to allow a more precise understanding of molecular and cellular phenotypes for drug response and may help to understand the role of *cky-1* fully. The identification of *cky-1* as a putative candidate offers new plausible hypotheses relevant to a resistant phenotype, whereby *cky-1* may act (1) during development to establish a neuronal architecture that is more tolerant to hyperexcitability, such as that caused by ivermectin, and/or (2) in response to ivermectin exposure by initiating transcription of downstream genes to modulate the excessive excitation/inhibition imbalance, thereby mitigating the lethal effect. These hypotheses will require further validation, aided in the first instance by identifying the downstream targets of *cky-1*. However, it is clear that the molecular mechanisms by which parasites develop ivermectin resistance are more complex than previously appreciated. Broader systems biology approaches are likely needed to understand the relationship between direct evidence of selection in the genome and the downstream transcriptional responses that enable parasite survival when challenged with ivermectin. By defining the genomic landscape of anthelmintic resistance even in a single resistant strain, our results refocus effort away from candidate genes with limited support and redefine our understanding of the evolution of anthelmintic resistance in helminths of veterinary and medical importance.

### Limitations of the study

We chose two strains of *H. contortus* that were both genetically and phenotypically distinct from each other to initiate the genetic cross. However, the high degree of genetic variation between these strains made it challenging to identify causal variants from associated linked genetic variation, particularly using a pooled sequencing and X-QTL analysis framework. This framework was also potentially limited depending on the genetic architecture of the drug response; the single prominent peak associated with benzimidazole resistance was clear, however, it was not possible to determine the independence or the relative effect size of multiple QTLs that were different in both height and breadth, such as those observed in the levamisole or ivermectin analyses. Advances in single worm phenotyping and sequencing will enable these multigenic signatures to be better defined and greater statistical power to differentiate causal from linked variants.

## STAR★Methods

### Key resources table

**Table:** 

REAGENT or RESOURCE	SOURCE	IDENTIFIER
**Bacterial and virus strains**		

*Escherichia coli* OP50	*Caenorhabditis* genetics Center (CGC)	OP50-1

**Chemicals, peptides, and recombinant proteins**		

Ivermectin	Oramec Drench, Boehringer Ingelheim Animal Health, UK	MSORA01
Ivermectin aglycone	Santa Cruz Biotechnology	CAS 123997-59-1
Fenbendazole	Panacur 10% Oral Suspension, MSD Animal Health	35,455
levamisole hydrochloride	Levacide Low Volume, Norbrook	NBLEV11
Amphotericin B	Thermo Fisher	15290018
Lugol’s iodine stain	Thermo Fisher	12996307
RNAse A	Thermo Fisher	EN0531
Phenol/chloroform/isoamyl alcohol (25:24:1)	Thermo Fisher	15593031
EB buffer	Qiagen	19086
L4440 double T7 RNAi expression vector	Andrew Fire; Addgene plasmid #1654; http://n2t.net/addgene:1654	RRID: Addgene_1654
Spermidine	Sigma	S2626
Proteinase K	Thermo Fisher	25530031

**Critical commercial assays**		

DrenchRite larval development assay, 96 well plate (containing ivermectin, levamisole and thiabendazole)	N/A	Gill et al., 1995
SuperScript® III First-Strand Synthesis System	Thermo Fisher	18080051
Brilliant III Ultra Fast SYBR QPCR Master Mix	Agilent Technologies	600882
Ambion Megascript T7 transcription kit	Thermo Fisher	AM 1333
PCR cleanup kit	Qiagen	28,104
dsRNAi Megaclear kit	Ambion	AM1908

**Deposited data**		

*Haemonchus contortus* reference genome	https://parasite.wormbase.org/Haemonchus_contortus_prjeb506/Info/Index/	(Doyle et al., 2020)
Raw sequencing data	ENA	PRJEB4207
Curated data (vcfs, fst, allele frequency data)	ftp://ngs.sanger.ac.uk/production/pathogens/sd21/hcontortus_xqtl	

**Experimental models: Organisms/strains**		

*Haemonchus contortus* MHco3(ISE)	Moredun Research Institute, UK	N/A
*Haemonchus contortus* MHco18(UGA)	Moredun Research Institute, UK	N/A
*Haemonchus contortus* MHco4(WRS)	Moredun Research Institute, UK	N/A
*Haemonchus contortus* MHco10(CAVR)	Moredun Research Institute, UK	N/A
*Teladorsagia circumcincta* MTci2	Moredun Research Institute, UK	N/A
*Teladorsagia circumcincta* MTci5	Moredun Research Institute, UK	N/A
*Caenorhabditis elegans* N2	*Caenorhabditis* Genetics Center (CGC)	N2
*Caenorhabditis elegans* VC2274 [cky-1(gk1011) V/nT1 [qIs51] (IV;V)]	*Caenorhabditis* Genetics Center (CGC)	VC2274

**Oligonucleotides**		

Ce_CKY1_299bp_RNAi_F_Sac1: CGG AGCTCTGCAGATGTCATCTCCAAT	This paper	N/A
Ce_CKY1_299bp_RNAi_R_Xba1: GCTCT AGACTGTCGTTTTGCAGTTCTTGC	This paper	N/A
HCON_00135080_F1: CCAG TTGGTGACGATTCC	This paper	N/A
HCON_00135080_R1: GGG TTTGCTGGAGATGACG	This paper	N/A
HCON_00155390_F1: CCGAG ACCAGATCAATGTCG	This paper	N/A
HCON_00155390_R1: CACA CTGTCTTCGGCTATCG	This paper	N/A
TCIRC_GAPDH1_F: TTGA GAAACCAGCTAGCATGGA	Baker et al., 2011	N/A
TCIRC_GAPDH1_R: CGC ACCCTCCGAAGCA	Baker et al., 2011	N/A
TCIRC_CKY1_F: TCAG CGATGGCAATGGAAG	This paper	N/A
TCIRC_CKY1_R: GAAGG ACCGCCAAAAAGAG	This paper	N/A

**Software and algorithms**		

Fastqc	http://www.bioinformatics.babraham.ac.uk/projects/fastqc/	
MultiQC	Ewels et al., 2016	v.1.8
Trimmomatic	Bolger et al., 2014	v.0.32
Bwa	Li, 2013	v.0.7.12-r1039
Picard	https://github.com/broadinstitute/picard	v.2.5.0
Samtools	Li et al., 2009	v.1.3
GATK	McKenna et al., 2010	v.3.7.0
Bcftools	Li et al., 2009	v.1.9
SNPeff	Cingolani et al., 2012	v.4.3r
Npstat	Ferretti et al., 2013	v.1
Popoolation2	Kofler et al., 2011	v.popoolation2_1201
vcftools	Danecek et al., 2011	v0.1.16
Mafft	Katoh and Standley, 2013	v7.407
I-TASSER	Yang et al., 2015	https://zhanggroup.org/I-TASSER/
Custom scripts	This paper	Github: https://github.com/stephenrdoyle/hcontortus_XQTL Zenodo: https://doi.org/10.5281/zenodo.6582305

### Resource availability

#### Lead contact

Further information and requests for resources and reagents should be directed to and fulfilled by the lead contact, Stephen Doyle (stephen.doyle@sanger.ac.uk).

#### Materials availability

All requests for resources and reagents should be directed to the lead contact author.

### Experimental model and subject details

#### Ethics statement

All experimental procedures were examined and approved by the Moredun Research Institute Experiments and Ethics Committee and were conducted under approved UK Home Office licenses following the Animals (Scientific Procedures) Act of 1986. The Home Office licence number is PPL 60/03899, and the experimental code identifier is E46/11.

#### Sheep

Sheep used for experimental infections of *H. contortus* were born and raised under clean conditions at the Moredun Research Institute, UK. The recipient sheep requiring surgery to initiate the genetic cross required anesthesia, which was induced using either intravenous thiopentone injection or a halothane and oxygen mask, after which the sheep was intubated, and anesthesia was maintained using halothane and oxygen. During the procedure, the sheep was routinely injected with a non-steroidal anti-inflammatory agent (1 mg/kg meloxicam; Metacam 20 mg/mL solution for injection; Boehringer Ingelheim) and antibiotic (7 mg/kg amoxicillin/1.75 mg/kg clavulanic acid, Synulox ready-to-use injection; Pfizer) and closely monitored on completion of the surgery. No adverse effects were noted. Sheep were humanely killed under schedule 1 of the Animals (Scientific Procedures) Act.

#### Parasitic nematode strains

This primary experiment of this study describes the analysis of a genetic cross between the anthelmintic susceptible strain MHco3(ISE) (Redman et al., 2008) and MHco18(UGA), a field-derived strain of *H. contortus* that is insensitive to standard treatment doses of benzimidazoles, levamisole, and ivermectin (Williamson et al., 2011). The MHco3(ISE) is derived from the SE strain, of which the precise history is unknown but is thought to have been originally isolated from East Africa. The MHco18(UGA) strain was originally isolated from sheep at the University of Georgia (UGA) Sheep Unit in 2004 (Williamson et al., 2011). MHco10(CAVR) strain is derived from the Chiswick Avermectin Resistant (CAVR) strain, an ivermectin resistant field population originally isolated in Australia (Le Jambre et al., 1995) and the MHco4(WRS) strain is derived from the ivermectin resistant White River Strain (WRS) originally isolated from South Africa (van Wyk and Malan, 1988). *Teladorsagia circumcincta* strains MTci2 and MTci5 were used to validate the association between *cky-1* expression with ivermectin resistance; MTci2 is a drug-susceptible strain originally isolated in 2000 from the Central Veterinary Laboratories, Weybridge, UK, whereas MTci5 is a multi-drug resistant isolate—resistant to benzimidazoles, levamisole and ivermectin (Bartley et al., 2004; Skuce et al., 2010)—isolated from a UK field population in 2002 following the establishment of multi-drug resistance on the farm (Sargison et al., 2001). All *H. contortus* and *T. circumcincta* strains used in this study are cryopreserved at the Moredun Research Institute, UK.

### Method details

#### Establishment of the genetic cross between a susceptible and multi-drug resistant strain of Haemonchus contortus

We crossed females of the fully drug-susceptible MHco3(ISE) isolate of *H. contortus* with males of the triple-resistant MHco18(UGA) isolate *in vivo*. Briefly, two sheep were orally infected with *H. contortus* third-stage infective larvae (L_3_); one with 10,000 L_3_ of the MHco3(ISE) isolate and one with 10,000 L_3_ of the MHco18(UGA) isolate. Immature adult worms were recovered from both sheep at necropsy on day 14 post-infection. After being rinsed in physiological saline at 37°C and sexed based on morphology, 100 female MHco3(ISE) and 100 male MHco18(UGA) worms were surgically transferred into the abomasum of a third sheep. Feces were collected from day three post-transfer, and F1 eggs were coprocultured for 14 days to develop, after which the Baermann technique was used to recover the L_3_ larvae. The success of the cross was confirmed by the production of eggs representing the F1 generation by MHco3(ISE) females, the development of the F1 eggs to viable infective (L_3_) larvae (validated by whole-genome sequencing; (Doyle et al., 2018)), and the establishment of fertile adult F1 in a donor sheep. Larvae were passaged without drug selection to generate the F2 and all subsequent generations of the genetic cross. A fourth sheep was orally infected with ∼5,000 F1 L_3_, and from day 21 post-infection, F2 eggs were collected and cultured to L_3_ as above.

For all generations of the genetic cross, the L_3_ were either maintained in tap water at 8°C or exsheathed and snap-frozen in liquid nitrogen and stored for future infections. Adult worms were recovered at necropsy, rinsed, sexed and snap-frozen in batches of 20 males or females before archiving at −180°C.

To assess drug efficacy in the F2 adults used for X-QTL analysis, fecal egg counts were undertaken throughout the infection. Although there were insufficient sheep to undertake a full fecal egg count reduction test or controlled efficacy test, fecal egg counts for the three sheep in each group were compared pre-treatment (on day 35) and 7 or 14 days post-treatment for fenbendazole/levamisole and ivermectin, respectively. Based on the reduction in egg output, the F2 generation contained adult parasites resistant to standard doses of all three drugs, confirming the establishment of a stable triple-resistant genetic cross. However, the resistance level differed greatly between drugs; fenbendazole and levamisole showed 73% and 94% efficacy, respectively, while ivermectin showed 10% efficacy. These findings are broadly consistent with the predicted mode of inheritance of resistance to each drug, i.e., partially recessive for fenbendazole and levamisole (Sangster et al., 1998) and dominant or partially dominant for ivermectin (Doyle et al., 2019a; Le Jambre et al., 2000).

#### Anthelmintic selection on the F2 generation with three anthelmintics (X-QTL)

The core aim of the work described was to perform a drug selection experiment on adults from the F2 generation of the genetic cross, followed by X-QTL genetic mapping of drug-specific genetic loci on F3 progeny by whole genome sequencing and analysis. The experiments were performed in triplicate. Briefly, F2 larvae were used for oral infection of 12 donors, and eggs were collected from day 21 and cultured to L_3_. On day 35, donor sheep were treated with either (i) 0.2 mg/kg ivermectin, (ii) 7.5 mg/kg fenbendazole, (iii) 7.5 mg/kg levamisole hydrochloride, or (iv) left untreated as a control. Eggs were collected from all donors for 21 days post-treatment and cultured to L_3_ as described above. Larvae collected pre- and post-treatment and from time-matched controls were snap-frozen in batches of 200 L_3_ for DNA extraction. A follow-up experiment to improve the resolution of the QTL by increasing the recombinants per pool was performed; in this case, ∼5000 L_3_ were pooled for DNA extraction. On day 56 post-infection, all donors were euthanised, and adult worms were harvested and stored as described above.

#### F3 selection with ivermectin (advanced intercross)

We performed subsequent selection experiments to refine the QTLs from the primary X-QTL analyses. The advanced intercross experiment, which involved drug treatment at half standard dose followed by a subsequent double standard-dose treatment of ivermectin was performed on the F3 generation adults, after which the F4 progeny were collected for whole genome sequencing and analysis. The experiment was performed in triplicate. Briefly, pre-treatment F3 generation L_3_ from three control donors in the X-QTL selection experiment were pooled, and aliquots of ∼5,000 L_3_ were used to infect seven donors (four for IVM treatment, including one ‘test’ donor to ensure adult parasites survived the treatment regime, and three untreated controls). Eggs were collected from day 21. On day 28, four donors were treated with 0.1 mg/kg (half standard dose) ivermectin, and eggs were collected from all donors for the next seven days. On day 35, the test donor was treated with 0.4 mg/kg (double standard dose) ivermectin and continued to produce eggs over the following seven days. The remaining three donors on the drug treatment regime were given 0.4 mg/kg ivermectin on day 42. Eggs were collected for 14 days post-treatment from all donors. Eggs produced pre- and post-ivermectin treatment and from time-matched untreated controls were cultured to L_3_ and snap-frozen in batches of 200 larvae. On day 56, all donors were euthanised, and adult worms were harvested and stored as described above.

#### *In vitro* larval development and dose-response assays

We used the commercial DrenchRite larval development assay to quantify the effective concentration of anthelmintic that resulted in various proportions of the maximum effect of the drug (i.e. EC_50_ is the concentration to produce 50% of the maximum). The basis for the assay is the observation that eggs from drug-resistant populations hatch in the presence of ivermectin, levamisole or thiabendazole and develop to L_3_ over 6–7 days. In contrast, eggs from drug-sensitive populations fail to hatch (thiabendazole) or hatch then arrest at L1 or L2 (thiabendazole, levamisole and ivermectin) in a concentration-dependent manner. The proportion of eggs that develop to L_3_ across a range of increasing drug concentrations is used to generate a dose-response curve, from which the EC_50_ is used as a measure of the resistance status of the adult population (Gill et al., 1995).

We used this approach to: (i) determine the EC_50_ for the parental isolates and F3 generation of the genetic cross in order to quantify the resistance status of the parental and admixed parasites; and (ii) determine the EC_25_, EC_50_ and EC_75_ for the F5 generation of the genetic cross in order to define drug-susceptible and highly-resistant larvae for QTL mapping phenotypically.

The standard DrenchRite assay was performed as follows. Briefly, eggs were isolated from fresh feces using standard procedures and diluted to 10,000 eggs in 2 mL tap water with 22.5 μg/mL amphotericin B. The egg suspension was vortexed thoroughly, and 20 μL (∼100 eggs) was added to each well of a 96-well DrenchRite plate (Gill et al., 1995). Plates were sealed with parafilm and incubated at 26°C. After 24 h, when most eggs had hatched, 20 μL nutritive media (Earle’s salt solution [10% v/v], yeast extract [1% w/v], sodium bicarbonate [1 mM] and saline solution [0.9% sodium chloride w/v]) (Gill et al., 1995) was added per well. On day 6, the assay was terminated with Lugol’s iodine stain, and the contents of each well were transferred to flat-bottomed 96-well plates. The numbers of eggs, L_1_, L_2_ and L_3_ were counted per well using an inverted light microscope at 100–200× magnification. A Probit model was fitted to identify the EC_25_, EC_50_ and EC_75_ for ivermectin.

Next, to isolate large numbers of sensitive L_1_ at a low concentration of ivermectin and resistant L_3_ developing normally in the high concentration ivermectin conditions for QTL mapping, we developed an in-house “scaled-up” larval development assay for the F5 generation. To scale up the larval development assay, ivermectin aglycone was diluted in DMSO and added to 2% Nematode Growth Medium (NGM) agar without cholesterol to give final ivermectin concentrations that corresponded to those used in the DrenchRite assay: 3.9 nM (EC_25_), 15.6 nM (EC_50_) and 62.5 nM (EC_75_). The assay was performed in 12-well plates, and triplicate wells were prepared for each ivermectin concentration and DMSO controls. The experimental conditions of the scaled-up assay were as described above, except that 1000 eggs were added per well in 200 μL of tap water with amphotericin B, 200 μL nutritive media was added after 24 h, and the assay was stopped on day 5 (without iodine fixation) to retrieve sensitive L_1_ before degradation.

In our initial DrenchRite assays, the DMSO control wells typically showed between 75 and 95% of the population developing to L_3_, with a small proportion (5–25%) that would hatch but not develop normally. We reasoned that the L_1_ in the EC_25_ wells would also likely include a proportion of these hatched but non-viable larvae; given that we were trying to isolate truly susceptible parasites, it was important to distinguish these non-viable larvae from drug-susceptible larvae. However, because larvae could not be fixed before sequencing and high power microscopy was impractical at this scale. For this reason, sensitive L_1_ were isolated from the EC_50_ wells (defined as arrested L_1_ stages), and resistant L_3_ were isolated from the EC_75_ wells to separate the population for sequencing.

#### Sampling and dose-response of parasites from US farms

To complement the selection experiments derived from the genetic cross and X-QTL analyses, we sampled pools of L_3_ from nine farm populations in the US. These samples were collected as part of routine anthelmintic resistance screening and phenotyped for ivermectin, levamisole and thiabendazole resistance using the DrenchRite assay described above (Howell et al., 2008). See Table S2 for EC_50_ data for the three drug classes. These farms have applied different management strategies and drug exposure histories, and thus the worms will have been exposed to different drug selection pressure(s). While we do not have complete detail of the management history of these populations, we selected populations for comparative analysis from a larger collection of farms based on these DrenchRite EC_50_ data, from which an ivermectin susceptible, three moderately resistant and five highly resistant farm populations to ivermectin were chosen (see Figures S2 and S3). Due to the limited number of fully drug-sensitive populations from commercial farms, one additional drug-sensitive isolate from the University of Georgia was included (UGA-SUSC (George et al., 2018); Farm 1). The populations selected also have variable levels of thiabendazole and levamisole resistance; therefore, we used these populations to validate the candidate regions associated with all three drug classes that are likely to be under drug selection in the field.

Larvae were archived at −80°C in water or 70% ethanol, then thawed, rinsed in PBS and snap-frozen in batches of 200 L_3_ before DNA extraction.

#### Sample preparation and whole-genome sequencing

Genomic DNA was isolated from pools of 200 L_3_ or individual adult males as follows: 20 μL of 20 mg/μL proteinase K together with 300 μL lysis buffer (200 mM NaCl, 100 mM Tris-HCl, 30 mM EDTA pH 8, 0.5% SDS) was added to the frozen pellets of larvae or adult worms before incubation at 55°C for 2 h. Next, 10 μL of 10 mg/mL RNase A was added before incubation at 37°C for 10 min. Phenol/chloroform/isoamyl alcohol (550 μL; 25:24:1) was added to the lysate, shaken vigorously for 15 s, incubated at room temperature for 5 min, and then centrifuged at 14,000 g for 15 min at room temperature. The top layer was carefully removed to a fresh tube, and 0.1× volume sodium acetate pH 5.5 was added, followed by 3× volume 100% EtOH at room temperature, then 2 μL glycogen, before overnight incubation at −80°C. After 5 min centrifugation at 14,000 g at 4°C, the supernatant was carefully aspirated, and 500 μL 70% EtOH was added to the pellet before centrifugation for another 5 min at 14,000 g at 4°C. The supernatant was carefully removed over a lightbox to visualise the pellet before a brief spin to facilitate aspiration of any remaining EtOH. The pellet was air-dried until all traces of EtOH were removed before re-suspending in ≥10 μL EB buffer. Genomic DNA was stored at 4°C.

Individual sequencing libraries (mean length of ∼400 bp) were prepared using a PCR-free protocol as previously described (Kozarewa et al., 2009) and sequenced on Illumina HiSeq2500 (X-QTL & advanced intercross) and HiSeq4000 (US farm) platforms using 125 bp and 150 bp PE chemistry, respectively. We aimed to perform sufficient sequencing to achieve 100-fold coverage of the ∼283 Mb genome for each sample sequenced.

#### Mapping and variant calling

Raw sequence data were first assessed for quality using Fastqc (http://www.bioinformatics.babraham.ac.uk/projects/fastqc/) and visualised using MultiQC (Ewels et al., 2016). Reads were first trimmed using trimmomatic (version 0.32) (Bolger et al., 2014) before they were mapped to the *H. contortus* V4 reference genome (Doyle et al., 2020) (available here: https://parasite.wormbase.org/Haemonchus_contortus_prjeb506/Info/Index/) using BWA-MEM (bwa: version 0.7.12-r1039; (Li, 2013)) (parameters: -Y -C -M). Duplicate reads were marked using Picard (version 2.5.0; https://github.com/broadinstitute/picard), after which mapped paired reads were extracted using samtools (version 1.3) view (-f 14). Finally, indel realignment was performed using GATK (version 3.7.0) IndelRealigner (McKenna et al., 2010). Genetic variation was determined using samtools mpileup (parameters: -F 0.25 -d 500 -b bamlist), which was performed for each experimental group of samples separately (i.e. each drug treatment group including all replicates). To determine the predicted functional consequence of individual variants, mpileup files were processed using bcftools (version 1.9; parameters: call -vm -Oz), after which annotation of the variant call format (VCF) file was performed using SNPeff (Cingolani et al., 2012) (version 4.3r; parameters: -no-intergenic -no-downstream -no-upstream). The current annotation GFF used to build the annotation database is available at WormBase ParaSite (release 17; https://parasite.wormbase.org/index.html) (Doyle, 2022).

#### Genome-wide analyses of genetic variation

To determine within sample measures of nucleotide diversity, multi-sample mpileup files were first processed to generate a single pileup file per sample per chromosome, which were subsequently used as input to npstat (version 1; parameters: -n 400 -L 5000 -mincov 20 -maxcov 200 -minqual 20 -nolowfreq 2) (Ferretti et al., 2013).

Between-sample measures of genetic diversity were determined using Popoolation2 (version popoolation2_1201) (Kofler et al., 2011); briefly, a synchronised file was first generated from each mpileup (popoolation2 mpileup2sync.jar --min-qual 20). Pairwise estimates of genetic differentiation were first determined by *F*_ST_ calculated in 5 kb windows throughout the genome (popoolation2 fst-sliding.pl --pool-size 200 --window-size 1000 --step-size 500 --min-count 4 --min-coverage 50 --max-coverage 2%), and secondly using a Fisher’s exact test on individual variants (popoolation2 fst-sliding.pl --window-size 5000 --step-size 5000 --min-count 4 --min-coverage 30 --max-coverage 2%). To determine the statistical significance of the genetic differences between groups, the binomial distribution of the *F*_ST_ data was used to calculate a *Z* score, from which a p value could be derived. A normalised p value, or q value, was then calculated to account for multiple hypothesis testing.

Finally, a Cochran-Mantel-Haenszel (CMH) test of independence was undertaken to determine concordance of replicate samples per variable site (popoolation2 cmh-test.pl --min-count 2 --min-coverage 30 --max-coverage 2% --population).

Analyses of specific variants identified in the genome-wide analyses, including calculating allele frequencies and extracting annotated SNPs based on functional effect classes, were performed using vcftools (version 0.1.16) (Danecek et al., 2011).

#### Resolving pool-seq QTL: Recombination, haplotype blocks and linked variation

The experimental and analysis framework (genetic cross, X-QTL and pooled sequencing) presented here is an efficient way to measure genetic differentiation between two phenotypically different groups. However, the resolution to map causal loci using a genetic cross and X-QTL approach depends somewhat on the amount of recombination that has taken place and thus is a function of the number of recombinant individuals present. We assayed variation from 200 progeny per pool, a number somewhat arbitrarily chosen but one that we thought would allow sufficient recombinants and reproducibility between replicate experiments. Consequently, this partially limited our ability to refine the QTLs to relatively broad regions of genetic differentiation. Our ability to refine these QTLs to identify causal variants was further confounded by high levels of background (non-causal) genetic variation within the QTL linked to the causal variant and co-inherited from the founding resistant strain.

To estimate the degree to which the genetic cross and subsequent parasite passage enabled us to resolve QTLs, we evaluated the proportion and length of the parental haplotypes expected to be present in the sampled progeny used to define QTLs. This was defined in two ways: (i) per individual and (ii) per sampled population. The code describing this analysis is available here: https://github.com/stephenrdoyle/hcontortus_xqtl/blob/master/03_code/hcontortus_xqtl.workbook.haplotypeblocks.md.

The cross was established with 100 male and 100 female parasites. The F2 generation of the cross were the first recombinants produced between the two parental strains; ∼5000 F2 L3 were used to establish the generation undergoing drug treatment. The F3 generation sampled before and after drug treatment were also recombinant; in total, 400 F3 L3 (200 pre-drug treatment and 200 post-drug treatment) were sampled. From the *H. contortus* genetic map (Doyle et al., 2018), we expect 0.69 crossovers per chromosome on average (for the autosomes). Using the number of crossovers together with total recombinant progeny sampled, we estimate that ∼37,260 recombination events occurred; per individual, this equates to 93.15 recombination events, resulting in ∼5.1 Mb haplotype blocks of inherited linked variation, from the parental strains to the individual parasites sampled in the F3 generation. Taking account of the diploid genome size and the total number of recombinants, we further estimate that as a population of parasites in the F3 sample pool, the minimum block size of linked variation to be considered is 12,743.56 bp. Given that the *F*_ST_ was measured in 5 kb blocks, a signal of selection from a minimum of three adjacent blocks would be suggestive of linked selection expected under the cross design. This was important to define as it distinguishes robust signals from stochastically distributed individual windows that achieved genome-wide significance, which we conclude are non-specific signals. We independently estimated, using fenbendazole X-QTL *F*_ST_ data, the minimum number of adjacent windows required to reach genome-wide significance (defined as 3 standard deviations above genome-wide *F*_ST_ mean), with the null hypothesis that individual points above the threshold were randomly distributed throughout the genome, i.e., not in a QTL cluster of data points. Using a range of different numbers of adjacent windows (n = 1–10) and based on sampling under a Poisson distribution, we estimate three or more adjacent data points were required to achieve Bonferroni-corrected genome-wide significance at α = 0.01. Therefore, both approaches, estimated from the cross design and data, agree that a minimum of three adjacent data points achieving genome-wide significance reflects the minimum unit of a selection signal.

We note that these calculations are specific to the cross design presented and acknowledge that they are imprecise, given they are estimated from data we have not specifically generated to measure these parameters. However, they are useful for estimating some parameters in the cross. Further, they allow us to predict that increased resolution could be achieved by simply increasing the sample size per pool, as has been recently demonstrated using *C. elegans* in which millions of individuals were used per pool (17), and/or by assaying pools after a greater number of generations following the genetic cross. Further resolution toward defining causal loci could be achieved by reducing the genetic variation in the founding cross, either by crossing phenotypically distinct parental strains that are genetically more closely related or by experimental evolution whereby a resistance phenotype is gradually selected from a susceptible population using increasing drug exposure over time.

#### Resolving the impact of direct versus indirect sampling of drug treatment

The X-QTL analyses were performed on the progeny (environmentally accessible eggs developed to L_3_ in fecal cultures) of adult parasites exposed to anthelmintics *in vivo*. Therefore, measures of selection on genetic diversity were indirect; as the progeny sampled are not exposed to drug treatment, this approach assumes that the genetic variation sampled in the progeny accurately reflects the genetic variation in the adults directly exposed to drug treatment *in vivo*. Since the mode of inheritance of ivermectin resistance is likely dominant or semi-dominant (based on previous work in different *H. contortus* strains that share the same chromosome 5 QTL where F1 individuals are ivermectin resistant (Doyle et al., 2019a; Le Jambre et al., 2000)), mating between phenotypically-resistant heterozygous adults will produce ∼25% phenotypically susceptible, homozygous progeny. The consequence of this is a reduction in both: (i) the overall frequency of the resistant allele in the post-treatment progeny populations; and (ii) the genetic differentiation between pre- and post-treatment populations (i.e. the causative resistant allele will not go to fixation in the surviving, post-treatment progeny population due to the presence of phenotypically susceptible individuals). To address this, we performed two additional complementary experiments to test the effect of direct selection. First, we sequenced pools of F3 adult male worms that survived treatment and compared them against pooled control L_3_ from the same generation. The genome-wide genetic differentiation between the two groups was generally more variable (Figure S7A), perhaps due to: (i) the lower number of individuals sampled (n = 40 adult males), (ii) the comparison between single-sex adults and mixed-sex L_3_, or (iii) the comparison between parasite populations sampled from different hosts (as opposed to the X-QTL experiments in which the pre- and post-treatment were sampled from the same host). However, the chromosome 5 QTL was still discernible with the peak of differentiation between 37.3 and 37.5 Mb (Figure S7B). Second, we performed *in vitro* larval development assays on the F5 generation of the cross, where eggs were cultured to L_1_/L_2_ or L_3_ in a concentration gradient of ivermectin. We first performed a dose-response experiment to determine the EC_25_, EC_50_ and EC_75_ with the DrenchRite assay (Figure S7C). A larger-scale development assay followed this experiment at relevant concentrations to isolate large numbers of larvae for whole-genome sequencing. Two groups of larvae were obtained and analyzed: (i) those that were susceptible to low concentrations of ivermectin (15.6 nM and below) and arrested at the L_1_ stage, and (ii) those that were resistant to ivermectin and proceeded to develop at high concentrations (62.5 nM and above). Again, a clear QTL at the chromosome 5 locus with comparatively little background variation was observed between the two groups (Figure S7D).

#### Analyses of acr-8 variation

We explored variation in *acr-8*, a gene previously implicated in levamisole resistance and a candidate that we identified in our X-QTL analyses of levamisole selection, in several different ways. First, we calculated the frequency of the *acr-8* indel, a marker previously associated with resistance, by counting sequencing reads containing the deletion pre-to post-treatment based on sequencing read coverage within and outside the indel for each replicate condition. Second, upon the identification of the Ser168Thr variant as being highly associated with resistance, we compared the protein sequence conservation of ACR-8 among clade V nematodes by aligning protein sequences obtained from WormBase Parasite using mafft (version 7.407) (Katoh and Standley, 2013), which were visualised using the ggmsa (https://cran.r-project.org/web/packages/ggmsa/vignettes/ggmsa.html) R package. Finally, we explored the putative protein structure of ACR-8, again with particular reference to the position of the candidate levamisole-resistance associated variant Ser168Thr, using the I-TASSER (Iterative Threading ASSEmbly Refinement) webserver (https://zhanglab.dcmb.med.umich.edu/I-TASSER/) (Yang et al., 2015) and compared predictions against known molecular structures at the Protein DataBank.

#### Real-time quantitative PCR (RT-qPCR) of cky-1

To investigate functional changes at the *cky-1* locus, its expression was determined in ivermectin susceptible and resistant *H. contortus* isolates. Total RNA was extracted from 20 male worms from each of three different donor sheep per isolate (MHco3[ISE]), MHco18[UGA], MHco4[WRS] and MHco10[CAVR]). RNA (1 μg) was used for oligo(dT) primed cDNA synthesis (SuperScript® III First-Strand Synthesis System), with no-reverse transcriptase controls included for each sample. cDNA was diluted 1:10 for RT-qPCR and 1 μL added to each reaction. RT-qPCR was undertaken on triplicate samples following the Brilliant III Ultra-Fast SYBR QPCR Master Mix protocol using the Mx30005P system (Agilent), and results were analyzed with MxPro (version 4.10). Gene expression was normalised to *β-actin* (HCON_00135080). All primer sequences are listed in key resources table.

To test the ubiquity of the association with resistance, expression of *cky-1* was also assessed in *T. circumcincta*; the same methods were used, except total RNA was extracted from 20 male worms from the susceptible MTci2 and ivermectin resistant MTci5 *T. circumcincta* isolates, a 1:5 cDNA dilution was used, and gene expression was normalised to *gapdh-1* (Baker et al., 2011).

#### RNA interference (RNAi) of cky-1 in C. elegans

To explore the effect of *cky-1* expression on ivermectin response, we hypothesised that RNAi knockdown of *cky-1* expression would increase the sensitivity of *C. elegans* to ivermectin. A 299 bp fragment of *Cel-cky-1* was amplified by PCR (Pfu Ultra II Phusion polymerase) from adult *C. elegans* cDNA and purified using a PCR cleanup kit. This product was cloned into TOPO-TA 2.1 and Sanger sequenced (Eurofins) using T7 primers to confirm its identity and that the full-length sequence was present. It was then sub-cloned by SacI/XbaI restriction digest followed by ligation into the L4440 double T7 RNAi expression vector and sequenced again to confirm the construct.

Double-stranded RNA (dsRNA) was prepared from an L4440:Ce299bpCKY1 clone using the Ambion Megascript T7 transcription kit. The following mixes were prepared in 200 μL PCR thin-walled RNase- and DNase-free tubes: three tubes containing 2 μL of 5X soaking buffer (1.25X M9 Mg^2+^ free, 15 mM spermidine, 0.25% gelatin) and 8 μL of dsRNA CeCKY1 299 bp fragment; three tubes containing 2 μL 5X soaking buffer and 8 μL of elution buffer from the dsRNAi Megaclear kit.

*Caenorhabditis elegans* N2 (Bristol “wild-type” laboratory strain) worms were grown to early L_4_ stage on standard Nematode Growth Medium (NGM) plates containing *Escherichia* coli OP50. Before RNAi-treatment, individual worms were picked onto empty NGM plates (without adding OP50) and allowed to crawl for a few minutes to remove any residual OP50. Thirty L_4_ were transferred into the prepared RNAi or control tubes and incubated at 20°C for 24 h. Drug plates were prepared with DMSO or ivermectin to a final concentration of 0.5, 1.0 and 2.0 ng/mL ivermectin; a single treated worm from either RNAi or buffer-only tubes was transferred to each of 10 plates for all drug concentrations. The worms plates were then placed at 20°C for four days and assessed for development. All worms that reached the L_4_ stage and above were counted and recorded.

We also measured the effect of the reduced *cky-1* expression on ivermectin sensitivity using a balanced deletion line of *C. elegans*, VC2274 [cky-1(gk1011) V/nT1 [qIs51] (IV; V)]. Five L_4_ worms were transferred onto each of five plates and left at 20°C for five days. Additional worms and a longer assay duration were used due to the lower fecundity and slower development rate to adulthood of the VC2274 line relative to N2. The numbers of worms reaching L_4_ stage and above were counted. The data presented are from three separate experiments.

### Quantification and statistical analysis

Genetic differentiation between paired samples was measured using *F*_ST_. Statistical analysis of the *F*_ST_ data was performed by converting the data to a *Z* score, from which an FDR-normalised q value was determined. Genome-wide level of significance is defined as either a Bonferroni correction threshold (α = 0.05) for genome-wide p value data or mean *F*_ST_ + 3 standard deviations for genome-wide *F*_ST_ data. A Cochran-Mantel-Haenszel (CMH) test of independence was performed on X-QTL replicate SNP data. Statistical analysis and data visualisation were primarily performed using R version 4.0.3. Details of specific statistical tests used are described in the figure legends where relevant.

## Data Availability

• Raw sequencing data have been submitted to ENA and are publicly available as of the date of publication. The key resources table describes the project accession number; specific experimental data are described in Table S1. • Analysis scripts have been deposited at GitHub and are publicly available as of the date of publication. DOIs are listed in the key resources table. • Any additional information required to reanalyse the data reported in this paper is available from the lead contact upon request.

## Supplemental information

Document S1. Figures S1–S8

Table S1. Sequencing data used in this study, related to all figures describing sequencing data as described in STAR Methods

Table S2. Phenotypic testing of drug resistance to benzimidazole, levamisole, and ivermectin using the DrenchRite larval development assay on parasites collected from US farm population, related to Figures 3 and 5, and is described in more detail in the STAR Methods

Document S2. Article plus supplemental information
