# Supplementary material for: Genomic landscape of drug response reveals mediators of anthelmintic resistance
Source: Cell Rep. 2022 Oct 18;41(3):111522. doi: 10.1016/j.celrep.2022.111522 (PMC9597552; doi:10.1016/j.celrep.2022.111522)
Supplement: Document S1. Figures S1–S8 [file mmc1.pdf]

Cell Reports, Volume *41*

Supplemental information

Genomic landscape of drug response reveals mediators of anthelmintic resistance

Stephen R. Doyle, Roz Laing, David Bartley, Alison Morrison, Nancy Holroyd, Kirsty Maitland, Alistair Antonopoulos, Umer Chaudhry, Ilona Flis, Sue Howell, Jennifer McIntyre, John S. Gilleard, Andy Tait, Barbara Mable, Ray Kaplan, Neil Sargison, Collette Britton, Matthew Berriman, Eileen Devaney, and James A. Cotton

Supplementary Information

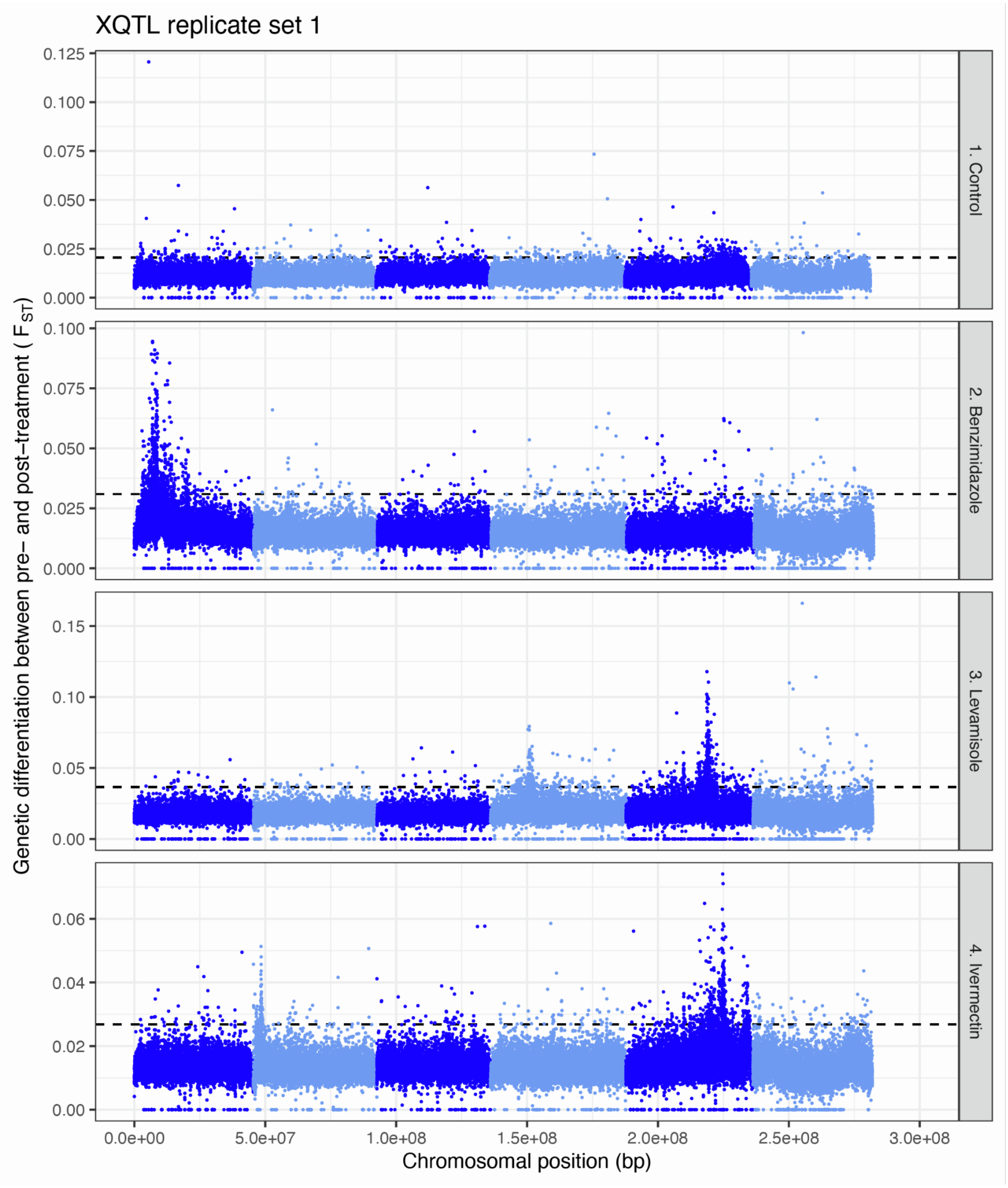
XQTL replicate set 1
Genetic differentiation between pre– and post–treatment (F_{ST})
Chromosomal position (bp)
1. Control
2. Benzimidazole
3. Levamisole
4. Ivermectin
0.0e+00
5.0e+07
1.0e+08
1.5e+08
2.0e+08
2.5e+08
3.0e+08

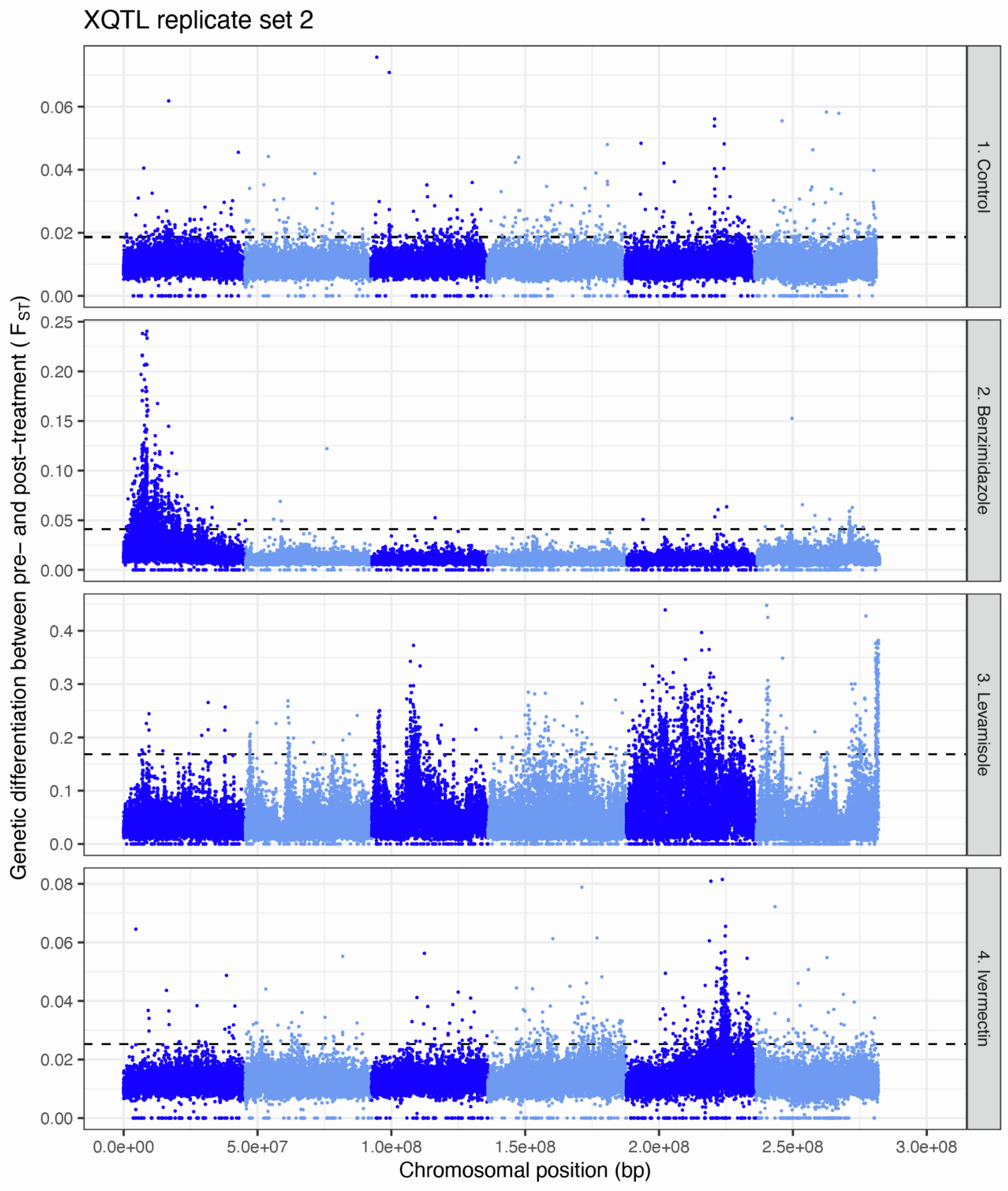

XQTL replicate set 2
1. Control
2. Benzimidazole
3. Levamisole
4. Ivermectin
Genetic differentiation between pre- and post-treatment (F_{ST})
Chromosomal position (bp)
0.0e+00
5.0e+07
1.0e+08
1.5e+08
2.0e+08
2.5e+08
3.0e+08

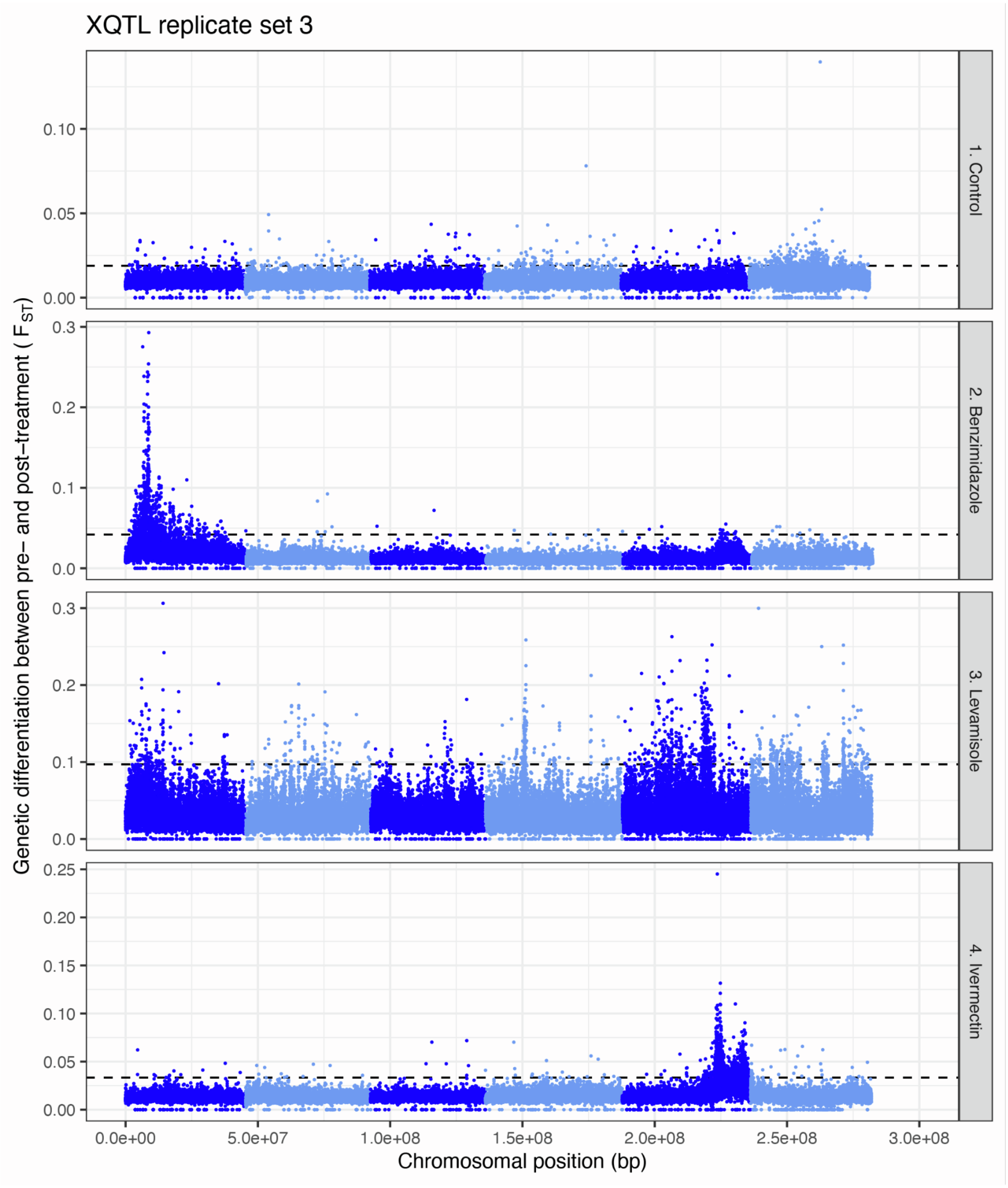

Figure S1. Related to Figure 2. Genome-wide differentiation (F_{ST}) between pre- and post-treatment populations of three independent replicate sets of the X-QTL experiment.

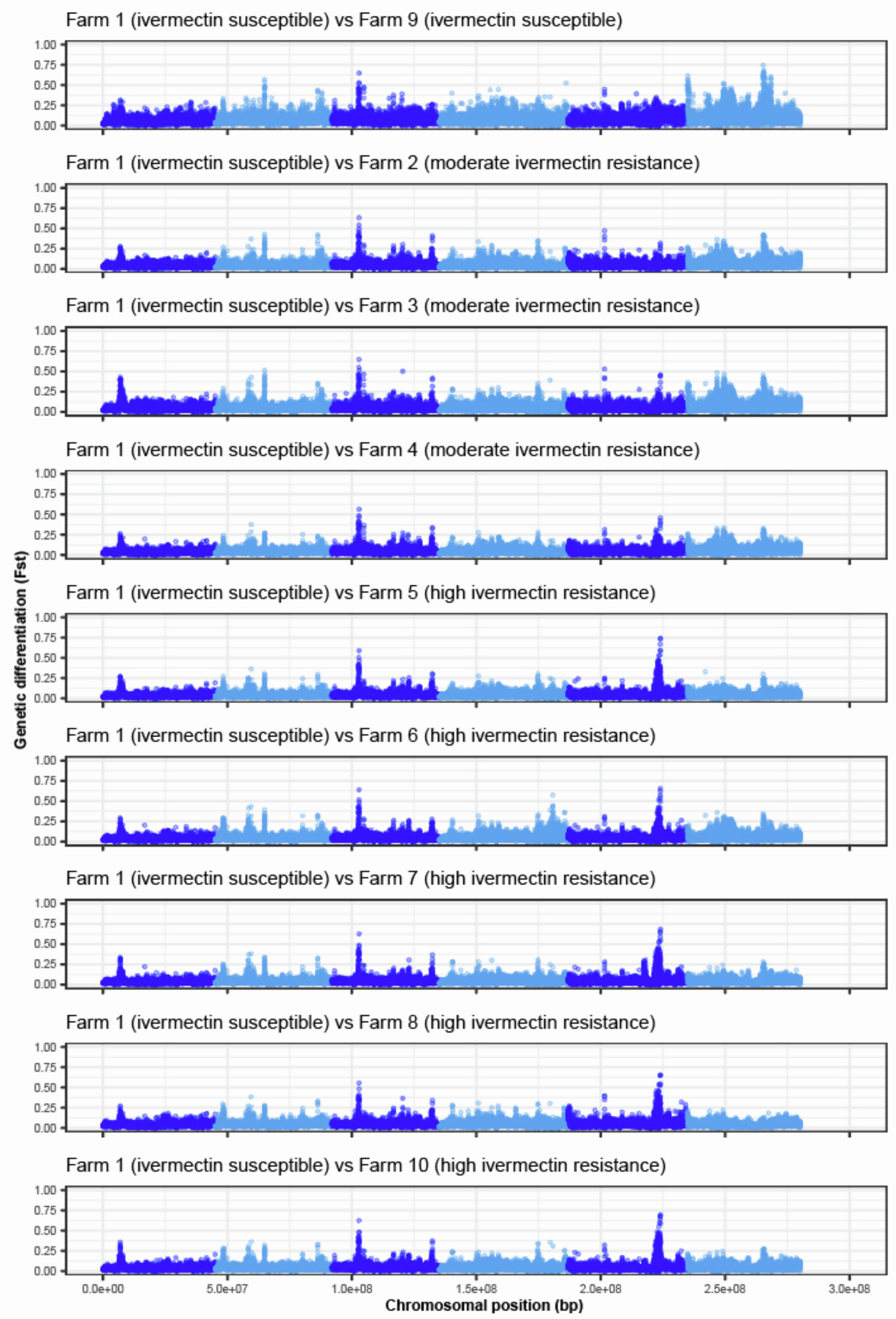

Figure S2. Related to Figure 3 and 5. Pairwise comparison of differentiation (F_{ST}) between a susceptible laboratory *H. contortus* isolate (Farm 1) and US farms that differ in sensitivity to ivermectin. Farm 1 is susceptible, and the rest have been classified as

moderately or highly ivermectin resistant based on the DrenchRite larval development assay.

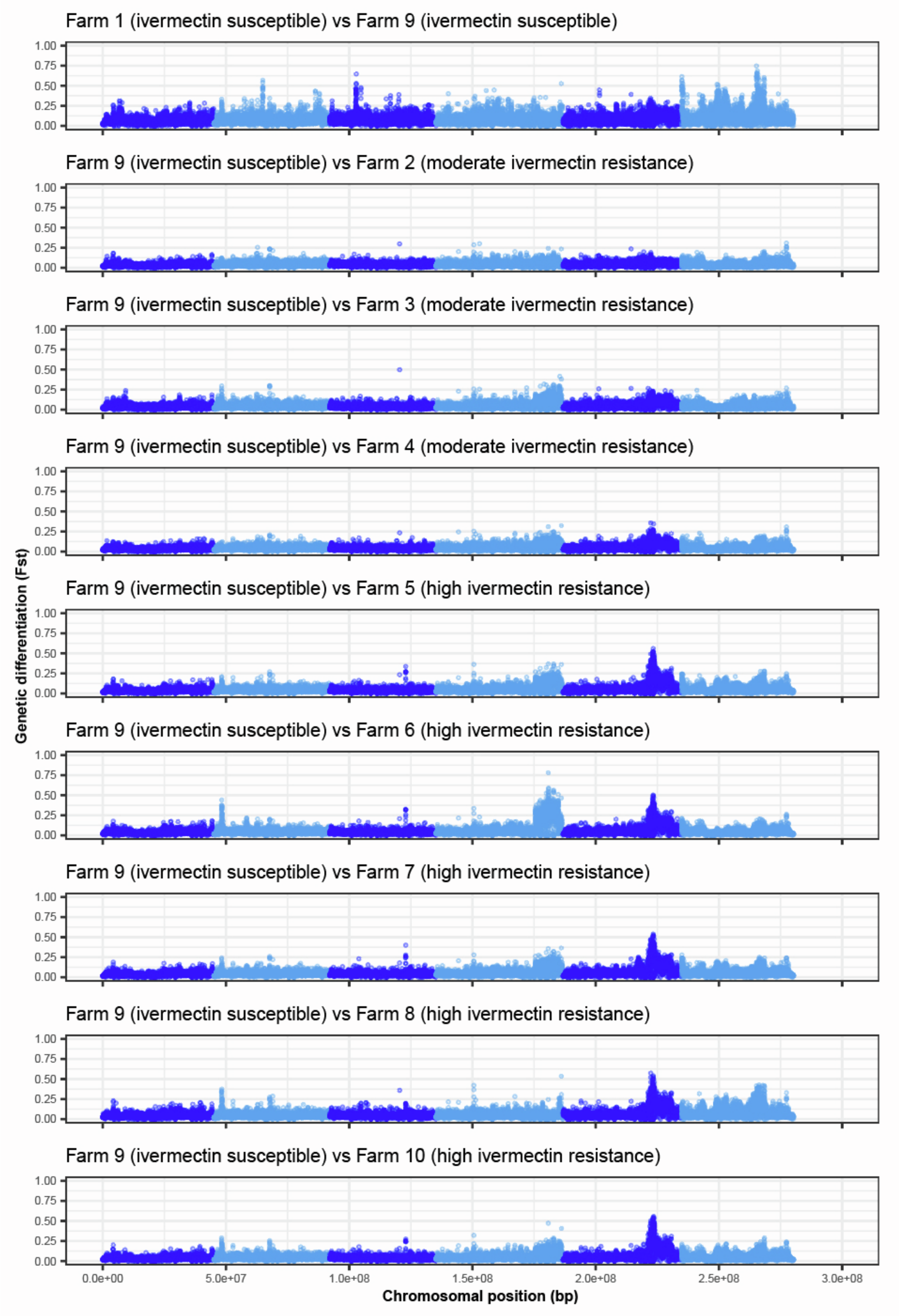

Figure S3. Related to Figure 3 and 5. Pairwise comparison of differentiation (F_{ST}) between a susceptible laboratory *H. contortus* isolate (Farm 9) and US farms that differ in

sensitivity to ivermectin. Farm 9 is susceptible, and the rest have been classified as moderately or highly ivermectin resistant based on the DrenchRite larval development assay.

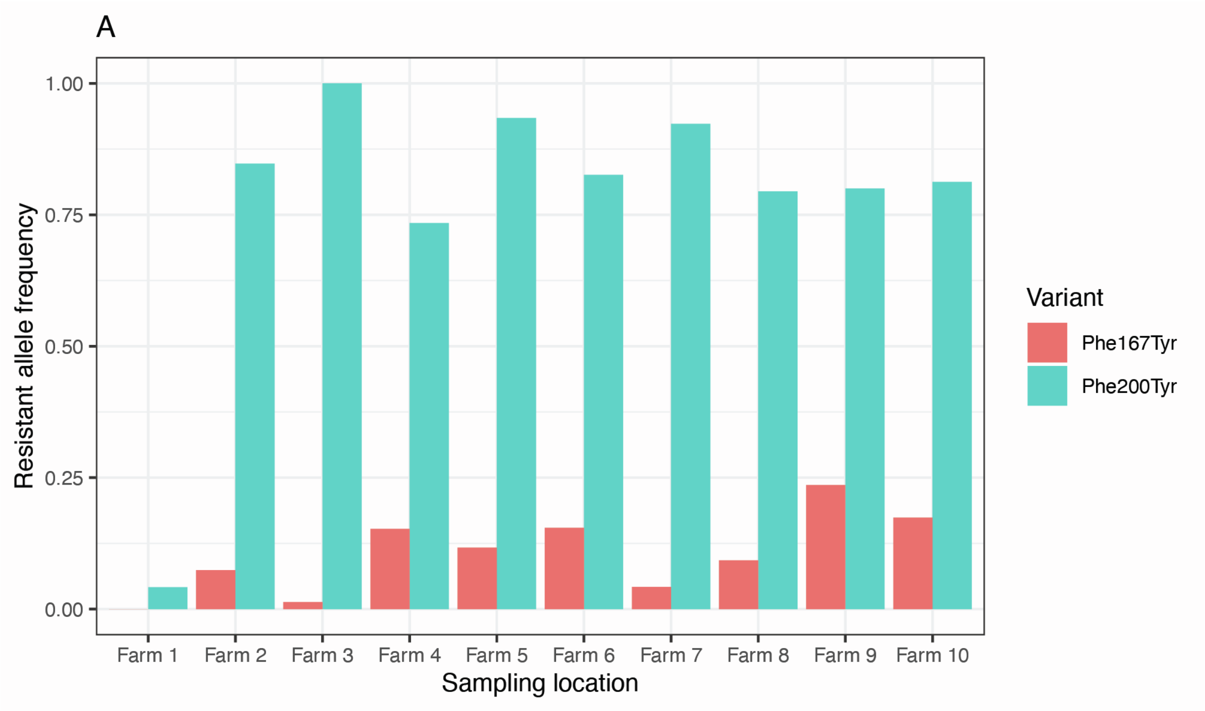

Figure S4. Related to Figure 3. Frequency of Phe167Tyr and Phe200Tyr variants of beta-tubulin isotype 1 associated with benzimidazole resistance from a US laboratory strain and 9 US farm isolates. The frequency of Phe167Tyr and Phe200Tyr variants were estimated using whole-genome sequencing data from *H. contortus* L_3 on 10 US farms. DrenchRite larval development assays demonstrate that worms from Farm 1 are susceptible to thiabendazole treatment, consistent with the absence of resistance-associated variants. However, all other farms are phenotypically resistant to thiabendazole treatment (**Table S2**).

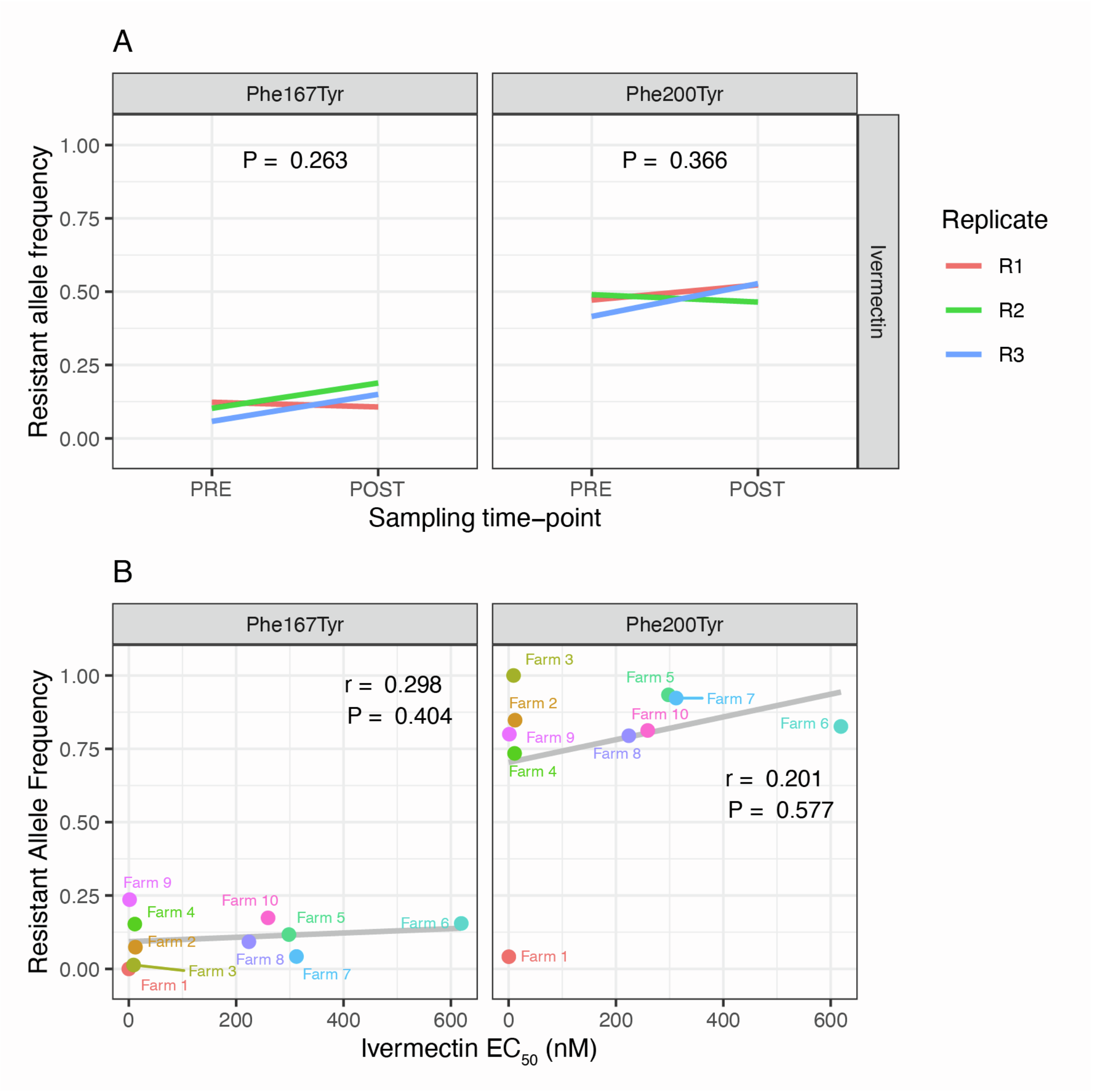

Figure S5. Related to Figure 3. There is no evidence of ivermectin selection on beta-tubulin isotype 1 resistant alleles in the genetic cross or US farms. (**A**) Estimation of Phe167Tyr and Phe200Tyr variant frequencies of *H. contortus* L_3 collected from pre- and post-ivermectin treated animals of the cross. Neither variant showed a significant change in variant frequency, measured using a paired t-test from which the *P*-values are shown. (**B**) Estimated Phe167Tyr and Phe200Tyr variant frequencies from *H. contortus* L_3 and their correlation with DrenchRite measurement of ivermectin resistance on 10 US farms. For both variants, no significant correlations were identified. Pearson's correlation (r) and associated *P*-value together with the linear regression trendline are shown.

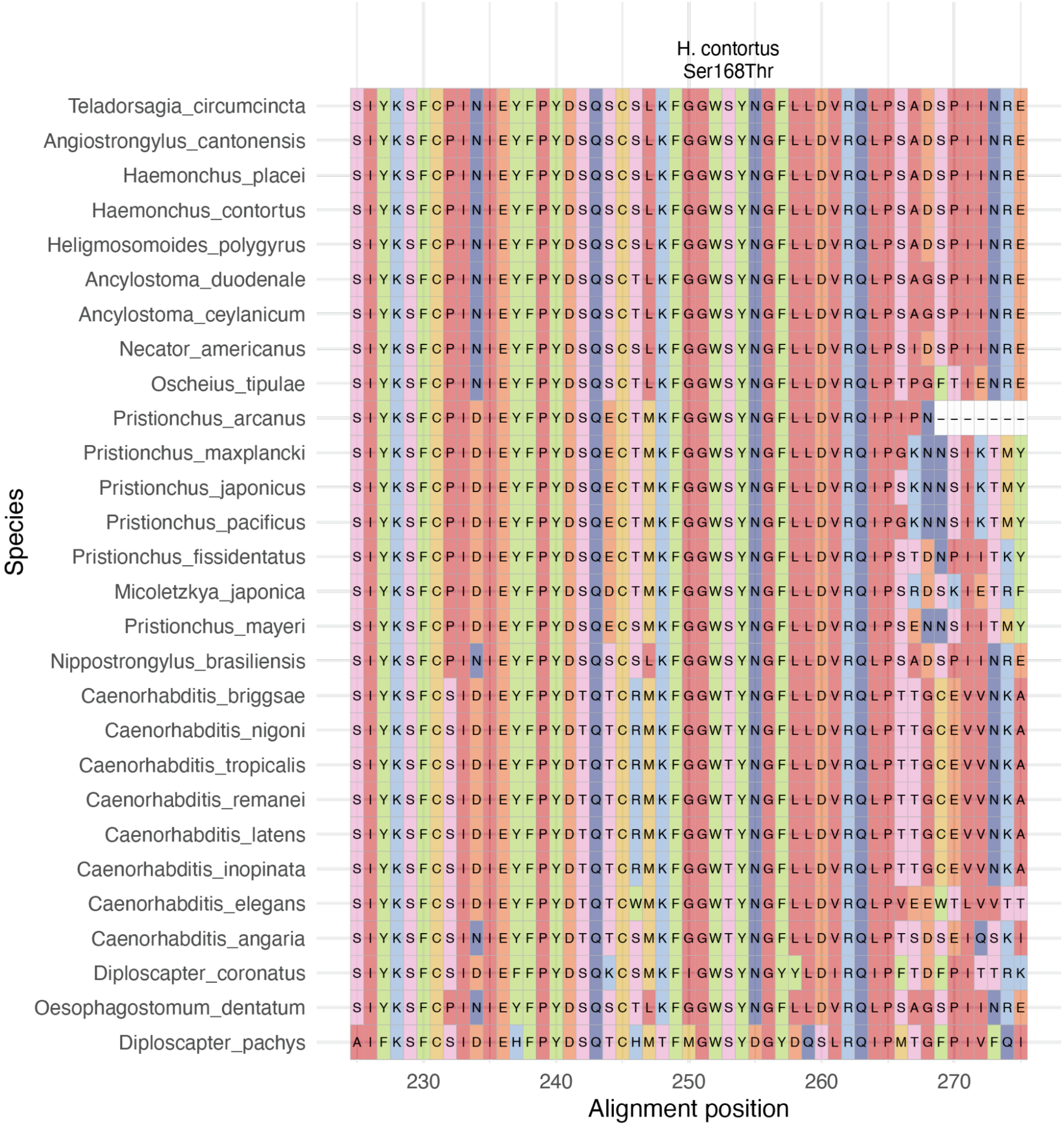

Figure S6. Related to Figure 4. Multiple sequence alignment of Clade V orthologs of acetylcholine receptor subunit ACR-8. A Ser167Thr variant in *Haemonchus contortus* (Ser position highlighted) is correlated with levamisole resistance. This multiple sequence alignment focused on alignment positions 225-275 surrounding the *H. contortus* Ser167Thr position demonstrates high sequence conservation of the Ser residue among all Clade V nematodes analysed, except for species within the *Caenorhabditis* genus (which contain Thr).

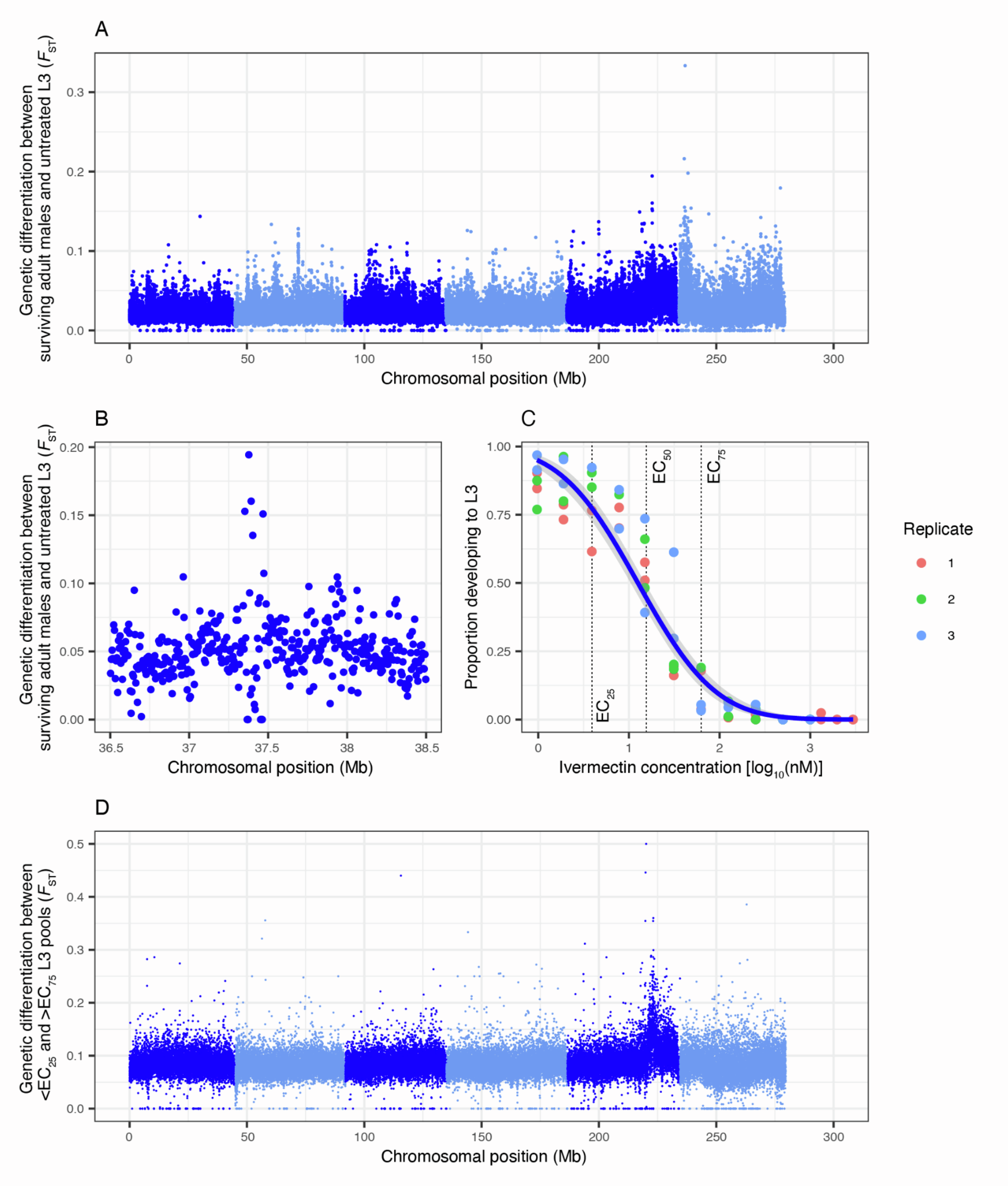

Figure S7. Related to Figure 5. Consistent evidence of selection on the chromosome 5 locus with respect to direct versus indirect measurements of genetic diversity after treatment with ivermectin. (**A**) Genome-wide analysis of genetic differentiation between pre-treatment L3 (n = 200; recovered from host faeces) and adult males (n = 40; recovered by host necropsy) directly exposed to and having survived treatment with ivermectin. (**B**) The same genomic interval on chromosome 5, as shown in Figure 5 A, highlights a smaller peak region. (**C**) Dose-response curve of ivermectin using larval development assays to determine the EC_{50}. (**D**) Genome-wide analysis of genetic differentiation between poorly developing L_1/L_2 stage larvae at EC_{25} and developing L_3 at EC_{75}.

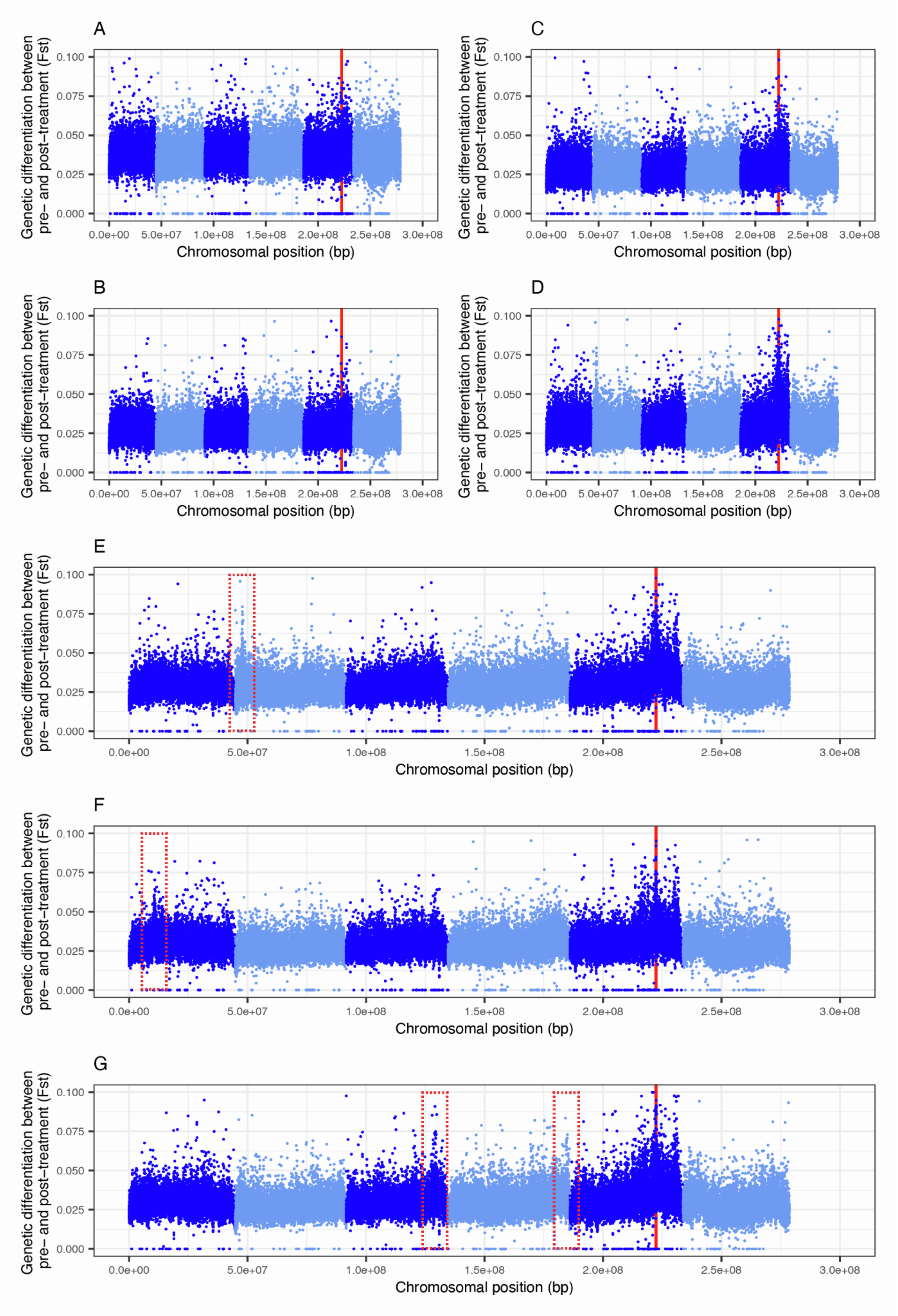
A
B
C
D
E
F
G
Genetic differentiation between pre- and post-treatment (Fst)
0.000
0.025
0.050
0.075
0.100
Chromosomal position (bp)
0.0e+00
5.0e+07
1.0e+08
1.5e+08
2.0e+08
2.5e+08
3.0e+08

Figure S8. Related to Figure 5. Differential dosing changes the genetic signature of ivermectin treatment. Comparison of untreated (**A**), time-matched control (**B**) and ivermectin treated populations that were sampled before and after the treated population were exposed to half-dose (**C**) followed by double dose (**D**) standard ivermectin treatment. When the experiment is replicated (each plot **E, F,** and **G** shows an individual replicate), new discrete QTLs are identified (red dashed boxes) in the double-dose treatment comparisons, in addition to the major QTL for ivermectin resistance on chromosome 5 (solid red line) that appears in all experiments in which ivermectin selection is applied.
